# Mitragynine, an euphoric compound inhibits hERG1a/1b channel current and upregulates the complexation of hERG1a-Hsp90 in HEK293-hERG1a/1b cells

**DOI:** 10.1038/s41598-019-56106-6

**Published:** 2019-12-24

**Authors:** Yea Lu Tay, Azimah Amanah, Mohd Ilham Adenan, Habibah Abdul Wahab, Mei Lan Tan

**Affiliations:** 1 Malaysian Institute of Pharmaceuticals & Nutraceuticals, NIBM, Ministry of Energy, Science, Technology, Environment and Climate Change (MESTECC), Pulau Pinang, 11700 Malaysia; 2 Atta-ur-Rahman Institute for Natural Product Discovery, Universiti Teknologi MARA (UiTM), Selangor Darul Ehsan, 42300 Malaysia; 3 School of Pharmaceutical Sciences, Universiti Sains Malaysia, Pulau Pinang, 11700 Malaysia; 4 Advanced Medical and Dental Institute, Universiti Sains Malaysia, SAINS@BERTAM, Kepala Batas, Pulau Pinang, 13200 Malaysia

**Keywords:** Toxicology, Cardiovascular biology

## Abstract

*Mitragyna speciosa* Korth (*M. speciosa*) has been widely used as a recreational product, however, there are growing concerns on the abuse potentials and toxicity of the plant. Several poisoning and fatal cases involving kratom and mitragynine have been reported but the underlying causes remain unclear. The human ether-a-go-go-related gene 1 (*hERG1*) encodes the pore-forming subunit underlying cardiac rapidly delayed rectifier potassium current (*I*_Kr_). Pharmacological blockade of the *I*_Kr_ can cause acquired long QT syndrome, leading to lethal cardiac arrhythmias. This study aims to elucidate the mechanisms of mitragynine-induced inhibition on hERG1a/1b current. Electrophysiology experiments were carried out using Port-a-Patch system. Quantitative RT-PCR, Western blot analysis, immunofluorescence and co-immunoprecipitation methods were used to determine the effects of mitragynine on hERG1a/1b expression and hERG1-cytosolic chaperones interaction. Mitragynine was found to inhibit the *I*_Kr_ current with an IC_50_ value of 332.70 nM. It causes a significant reduction of the fully-glycosylated (fg) hERG1a protein expression but upregulates both core-glycosylated (cg) expression and hERG1a-Hsp90 complexes, suggesting possible impaired hERG1a trafficking. In conclusion, mitragynine inhibits hERG1a/1b current through direct channel blockade at lower concentration, but at higher concentration, it upregulates the complexation of hERG1a-Hsp90 which may be inhibitory towards channel trafficking.

## Introduction

*Mitragyna speciosa* Korth from the Rubiaceae (coffee) family is a medicinal plant native to countries in South-East Asia. It is commonly known as kratom and is usually consumed as beverage or tea^1–4^. Kratom leaves have been used by natives to treat common illnesses and are popular for their energizing and pain alleviating effects. This plant is also known to possess psychostimulant- and opiate-like properties, depending on the dosage taken^1,3,5^. The narcotic, stimulant and other dose dependent effects of kratom have been attributed primarily to mitragynine^6^. Currently, kratom containing preparations in the form of crude extracts, dried leaf or resin, are widely available via the internet^7^. Kratom is generally consumed deliberately to gain the desirable effects of euphoria and to manage opiate withdrawal. Despite the wide range of pharmacological effects, studies into the toxicity related to kratom use are scarce. Clinical toxicological investigations were limited to case-reports involving the toxic effect of kratom extracts following short or long-term consumption^8^.

There have been several intoxications and fatal incidents involving kratom and mitragynine but the underlying causes were unclear. In most reports, co-administration with other medications or herbs were implicated^9–12^. For example, both mitragynine and O-desmethyltramadol were detected in blood samples of victims in a study reported in Sweden. Post-mortem analysis revealed that most of them developed lung edema and congestion^11^. Similarly, in another fatal kratom toxicity involving a young male, therapeutic levels of over-the-counter cold medications, benzodiazepines and mitragynine was detected simultaneously. Interestingly, the victim was also presented with pulmonary congestion and edema^13^. Unfortunately, the actual cause of death remained unknown and non-conclusive. Kratom has been reported to cause serious adverse effects, such as elevated blood pressure, nephrotoxic effects, impaired cognition and behavior and hepatic failure^2,7,14–16^.

Cardiac toxicity is one of the major reasons responsible for the suspension of preclinical or clinical drug discovery programs and the withdrawal of licensed drugs^17^. The risk of developing Torsade de Pointes (TdP), a lethal cardiac arrhythmia which is portrayed by long QT interval in electrocardiogram, has been a main reason for the removal of approximately 26% of post-marketed drugs between 1990 and 2005^18,19^. The human ether-a-go-go related gene (*hERG1*) channel, which is the pore-forming subunit underlying cardiac rapidly delayed rectifier potassium current (*I*_Kr_), is currently the most important target for cardiac safety evaluation^20,21^. Inhibition of *I*_Kr_ by a wide range of drug molecules can prolong repolarization of the cardiac action potential leading to serious complication, such as TdP and sudden cardiac death^22^. To date, most electrophysiological and pharmacological studies of *I*_Kr_ in respect of the heart have focused on the hERG1a isoform only^23–25^. However, several studies have confirmed that native *I*_Kr_ channels minimally compose of both hERG1a and hERG1b isoforms^26–31^. In heterologous expression systems, the hERG1a and hERG1b subunits co-assemble to form functional channels, and exhibit biophysical and pharmacological properties distinct from those of homomeric hERG1a channels and appear to better resemble those of native *I*_Kr_ current^29,30,32–34^.

Mitragynine is an euphoric agent and its abuse has been associated with serious side effects and death. Our previous study has reported that mitragynine blocks *I*_*Kr*_ in hERG1-transfected HEK293 cells and hERG1 cRNA-injected *Xenopus* oocytes, respectively. This study aims to further elucidate the molecular mechanisms of mitragynine inhibition on the hERG1a/1b channels and to support the thesis that mitragynine constitutes a cardiotoxicity risk.

## Results

### Electrophysiological properties of hERG1a/1b current in hERG1a/1b-transfected HEK293 cells

**Figure 1:**
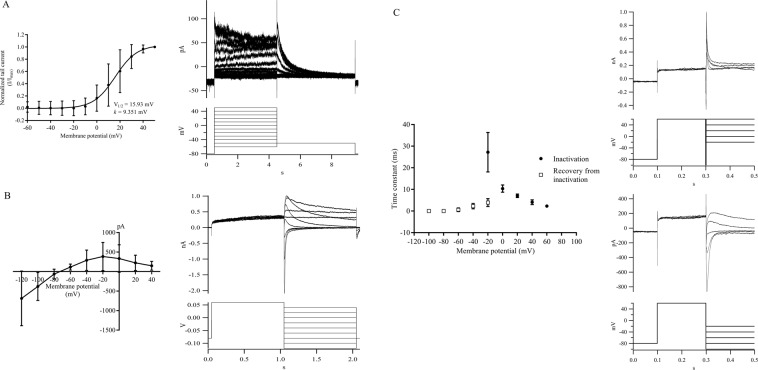
Electrophysiological properties of hERG1a/1b current in HEK293-hERG1a/1b cells. (**A**) Activation curve measured with hERG1a/1b tail currents and fitted to a Boltzmann relationship. Data are presented as mean ± SD of three independent experiments. (**B**) The fully activated I-V relation of hERG1a/1b current. Voltage clamp protocol and representative currents recorded from HEK293-hERG1a/1b cell. Data are presented as mean ± SD of six independent experiments. (**C**) Voltage dependence of the time constants for the development of inactivation (⦁) and recovery from inactivation (▫). Three-pulse and two-pulse protocols were used to study inactivation and recovery from inactivation properties of hERG1a/1b current and their representative currents. Data are presented as mean ± SD of three independent experiments. All measurements were carried out at room temperature.

The relative mRNA and protein expression of hERG1a/1b in HEK293-hERG1a/1b recombinant cells were verified prior to the patch clamp experiments. The mean mRNA expression of hERG1a and hERG1b in transfected HEK293 cells were approximately 400-fold (*p* < 0.0001) and 700-fold (*p* = 0.0003) higher than un-transfected HEK293 cells. On the other hand, hERG1a protein was identified by bands at 155 kDa [fully-glycosylated (fg), mature form] and 135 kDa [core-glycosylated (cg) precursor form], whereas hERG1b protein was identified by bands at 95 kDa (fg form) and 80 kDa (cg form), respectively (Supplementary Figs. 1–6). Figure 1A shows hERG1a/1b current recorded from a representative HEK293-hERG1a/1b cell at room temperature. During depolarizing steps, an outward current was observed at voltages positive to −20 mV, and the current amplitude reached a maximum at +40 mV. With further depolarization, the outward current amplitude decreased. Upon repolarization to −50 mV, tail current characterized by the “hook” was observed. The normalized tail currents were plotted against membrane potential and fitted to a Boltzmann function (Fig. 1A). The means of V_1/2_ and *k* were +15.93 mV and +9.351 mV respectively. The fully activated I-V relation for HEK293-hERG1a/1b is shown in Fig. 1B. Typical of hERG1 current, the currents showed inward rectification due to inactivation of the channels at more positive potentials. With repolarization to more negative voltages, hERG1a/1b current recovered from inactivation and subsequently underwent voltage-dependent decay. Maximum outward current was determined at −20 mV and at more negative voltages, the current became inward.

The inactivation and recovery from inactivation properties of hERG1a/1b are shown in Fig. 1C. The hERG1a/1b current was subjected to a depolarizing voltage step to +60 mV for 200 ms, followed by a brief hyperpolarizing step to −100 mV to allow the hERG1a/1b channels to recover from inactivation into the open state. The current amplitudes evoked by the test steps were relatively large and were rapidly inactivated when the voltages became more positive. The time constants of development of inactivation $({{\rm{\tau }}}_{{\rm{inact}}})$ were estimated by fitting the decay of the currents in the third pulse to a single exponential function, and the average data were plotted (Fig. 1C: filled symbols). The time constant of recovery from inactivation was determined using a two-pulse protocol. The cells were depolarized to +60 mV for 200 ms to activate and inactivate hERG1a/1b current, and subsequently repolarized to potentials between −20 and −100 mV to generate a tail current. This resulted in the rapid recovery of hERG1a/1b current which slowly decayed as channels deactivated (Fig. 1C). The time constant of recovery from inactivation was measured as the single exponential fit to the tail current initial rising phase (−40 mV to −20 mV). In potential ranges where deactivation is also present in the tail current (−100 mV to −60 mV), a double exponential fit was used to separate the two components (recovery from inactivation and deactivation). The time constant of recovery from inactivation was represented by the fast time constant (${{\rm{\tau }}}_{{\rm{fast}}}$). The mean time constants of recovery from inactivation are plotted as shown in Fig. 1C (open symbols). Both inactivation and recovery from inactivation displayed voltage-dependent characteristics and were depicted by a typical “bell-shaped” curve (Fig. 1C). Inactivation was the slowest at −20 mV (≈ 27.14 ms) and became faster with incremental increases in test potential. Similarly, recovery from inactivation was slowest at −20 mV (≈ 3.94 ms) and became faster with increasing hyperpolarization.

### The effects of mitragynine on cell proliferation

**Figure 2:**
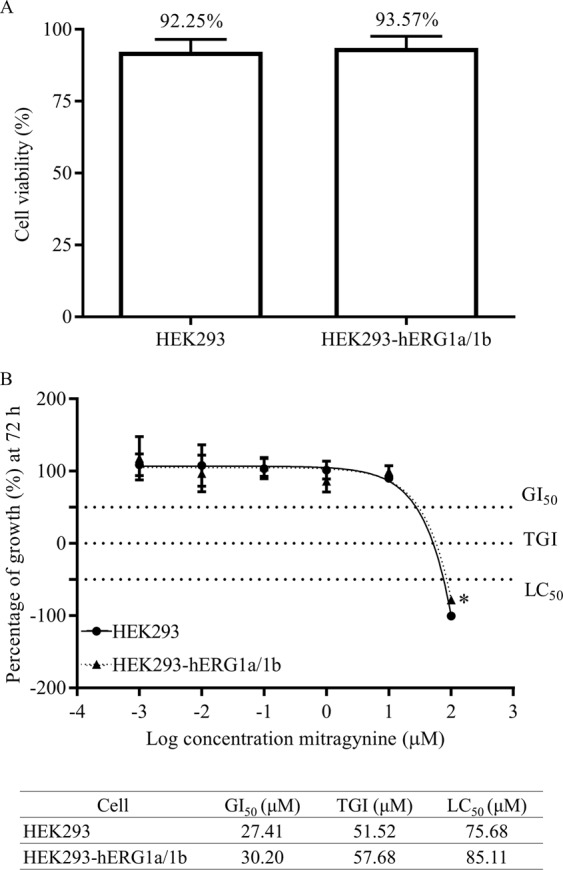
(**A**) Cell viability data for HEK293 and HEK293-hERG1a/1b cells. (**B**) Cell proliferation and cytotoxic parameters of mitragynine on HEK293 cells and HEK293-hERG1a/1b recombinant cells. Positive values (<100%) represent growth inhibition and negative values (<0%) represent cytotoxicity as compared with initial cells plated (T_0_). Data are presented as mean ± SD of three independent experiments. *p < 0.05 compared with HEK293 (unpaired t-test).

The cell viability of both un-transfected and transfected HEK293 cells (Fig. 2A) were 92.25 ± 4.28% and 93.57 ± 4.05% respectively. The percentage of cell viability between the two cells were similar (*p* = 0.7171), thus indicating that transfection did not affect cell viability. The stably transfected HEK293-hERG1a/1b cells were used for subsequent experiments. Figure 2B shows the cytotoxic parameters derived for mitragynine in HEK293 and HEK293-hERG1a/1b cells respectively. The percentage of growth and cytotoxic parameters of mitragynine in the recombinant cells appeared to be similar with the non-transfected cells. The GI_50_, TGI and LC_50_ values of mitragynine in HEK293-hERG1a/1b, were 30.2 µM, 57.68 µM and 85.11 µM respectively. Hence, test concentration ranges of mitragynine in subsequent experiments did not exceed the GI_50_ values (0.001–10 µM).

### The effects of control drugs and mitragynine on the hERG1a/1b current in HEK293-hERG1a/1b recombinant cells

**Figure 3:**
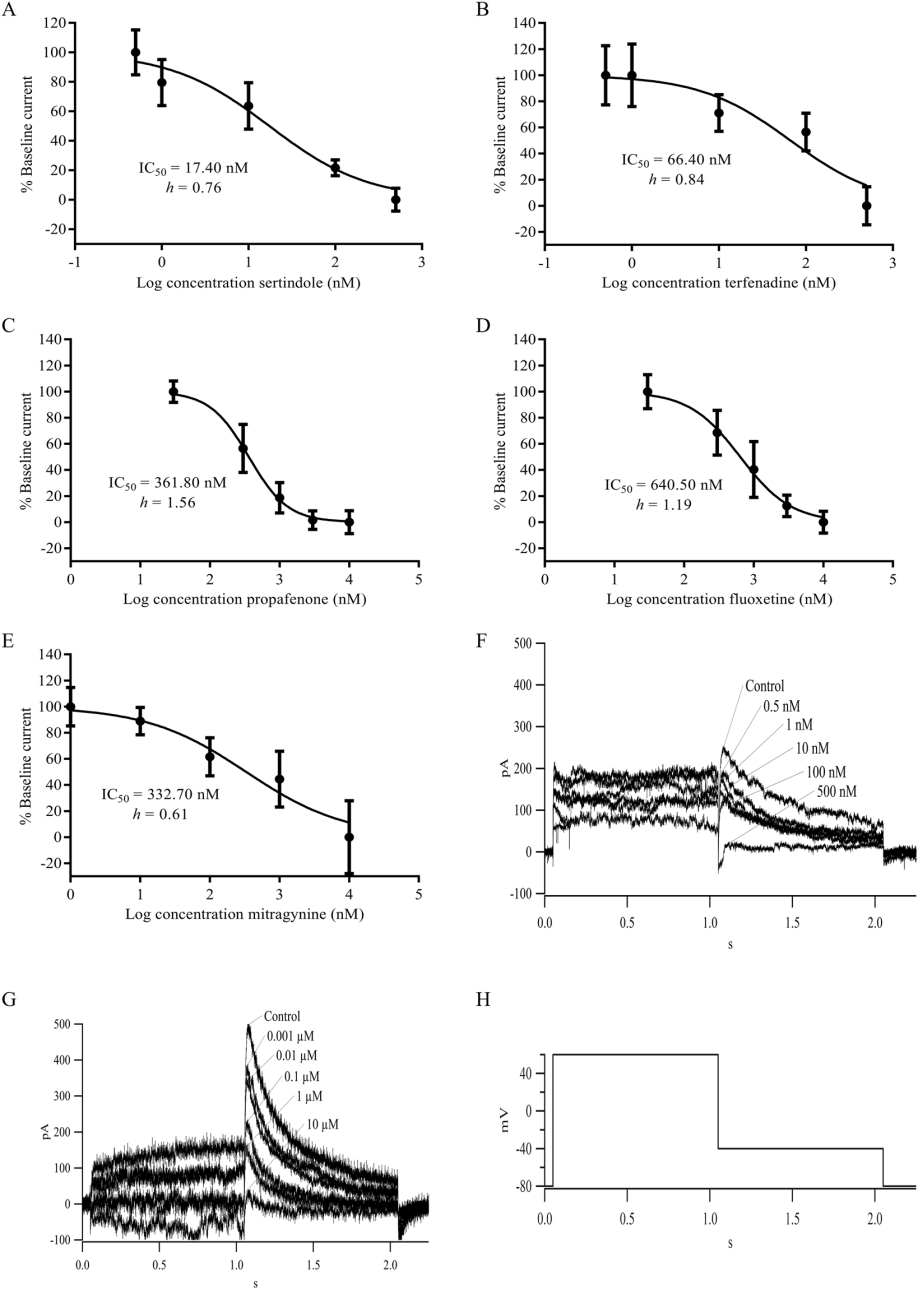
A non-linear regression and dose-response analysis of (**A**) sertindole, (**B**) terfenadine, (**C**) propafenone, (**D**) fluoxetine and (**E**) mitragynine on hERG current. Data represents mean ± SD of four independent experiments. Representative current recordings of concentration dependence inhibition of hERG1a/1b current by (**F**) terfenadine (**G**) mitragynine. (**H**) Step pulse inactivating protocol to elicit hERG tail current.

The effects of control drugs and mitragynine on hERG1a/1b current were evaluated using a step pulse protocol (Fig. 3A–H). In general, the currents detected were within the pA range. In this study, sertindole, terfenadine, propafenone and fluoxetine were used as positive controls for the patch clamp assays^35–38^. Representative traces from the hERG1a/1b current in the absence and presence of increasing concentrations of terfenadine and mitragynine are shown in Fig. 3F,G. The amplitude of the peak tail current decreased with increasing concentration of the positive control compounds indicating concentration-dependent inhibition of the peak tail current. Non-linear regression and dose-response analyses yielded IC_50_ values of 17.40 nM (*h* = 0.76), 66.40 nM (*h* = 0.84), 361.80 nM (*h* = 1.56) and 640.50 nM (*h* = 1.19) for sertindole, terfenadine, propafenone and fluoxetine respectively (Fig. 3A–D). Interestingly, mitragynine elicited a dose-dependent inhibition on the hERG1a/1b current with an IC_50_ value of 332.70 nM (*h* = 0.61) (Fig. 3E).

### Mitragynine significantly reduces fg-hERG1a but increases cg-hERG1a protein expression, similar with arsenic trioxide

**Figure 4:**
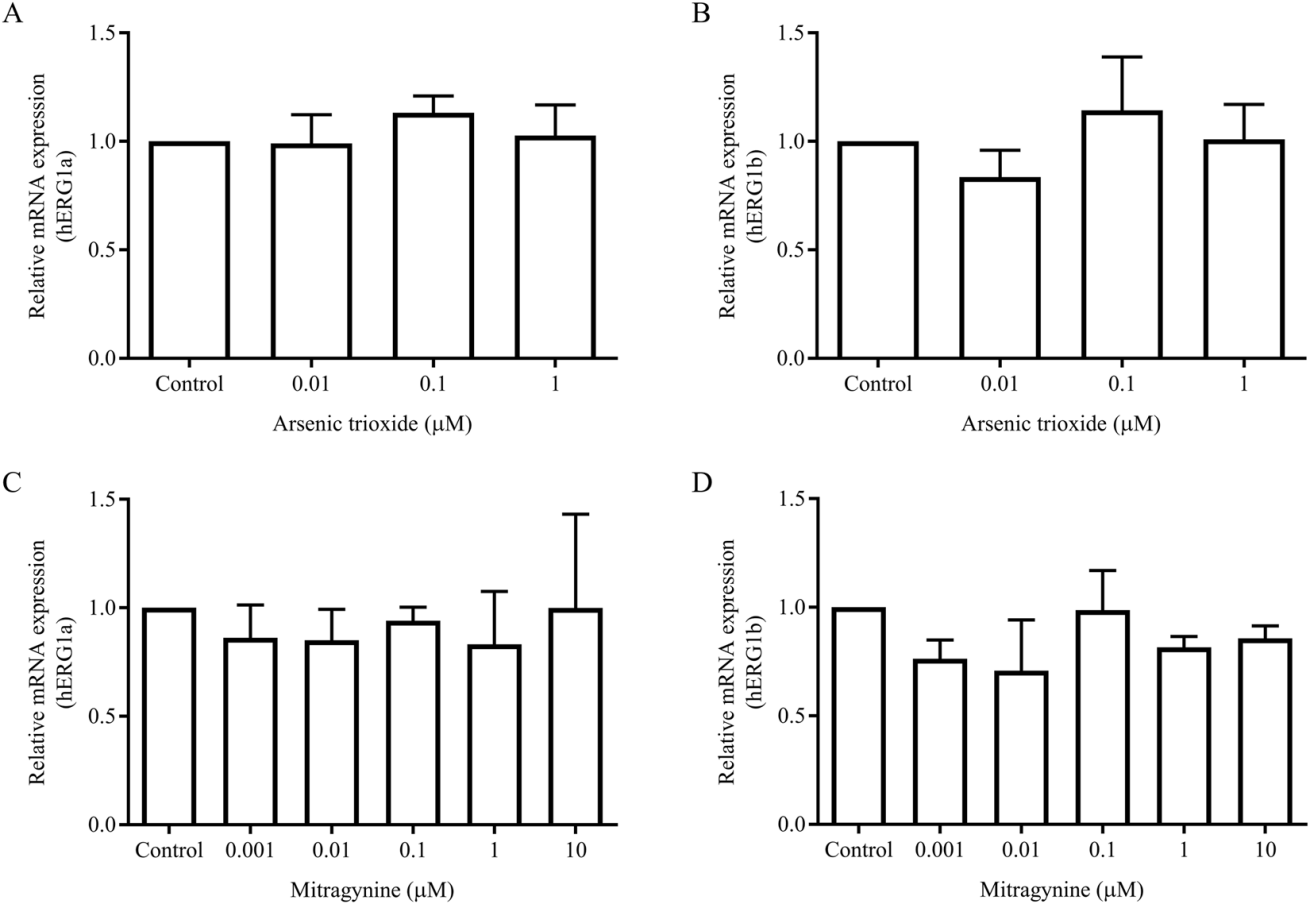
The effects of (**A,B**) arsenic trioxide and (**C,D**) mitragynine on mRNA expression of hERG1a and hERG1b after incubation for 24 h at 37 °C. Data are presented as the mean fold change ± SD of three independent experiments.

**Figure 5:**
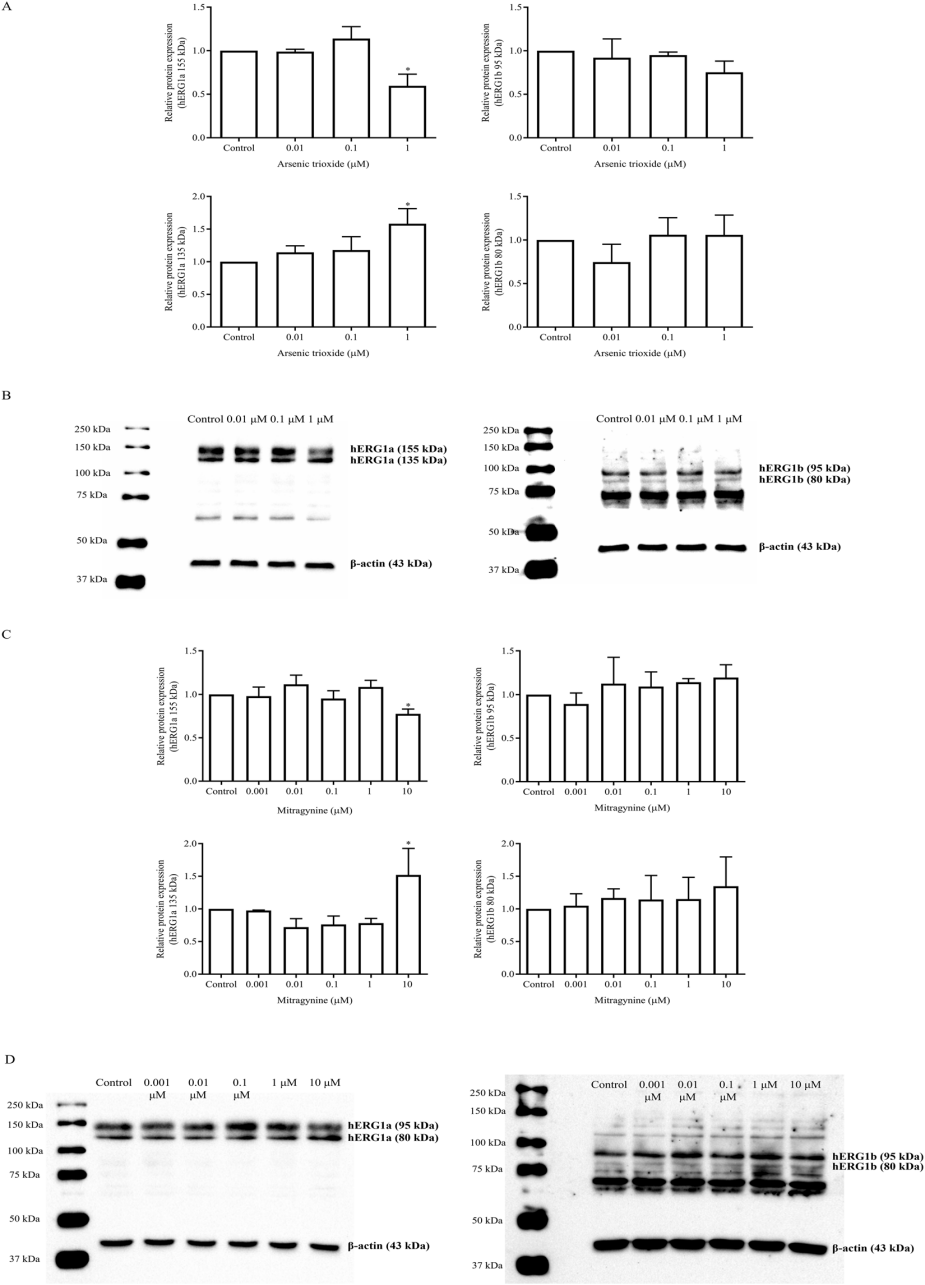
The effects of (**A,B**) arsenic trioxide and (**C,D**) mitragynine on the hERG1a/1b protein expression. Representative image of Western blot analysis for the protein expression of hERG1a (155 kDa and 135 kDa), hERG1b (95 kDa and 80 kDa) and β-actin (43 kDa) following treatment with arsenic trioxide or mitragynine for 24 h. Electrophoresis was carried out at 150 V for 90 min and the protein bands were then transferred to a single PVDF membrane at 1.0 A and 25 V for 30 min. The protein of interest and the internal control of protein loading, β-actin (43 kDa), were separated by cutting the membrane at around 50 kDa using the molecular weight marker as reference. The separated membranes were then placed in different containers and incubated with respective antibodies. After immunoblotting, the separated membranes were placed together and visualized using ChemiDoc^TM^ XRS Imaging System (Bio-Rad Laboratories Inc, USA). For (**B**) hERG1a & β-actin, the exposure time was 24 s, meanwhile for hERG1b & β-actin, the exposure time was 111 s. As for (**D**) hERG1a & β-actin, the exposure time was 16 s, meanwhile, for hERG1b and β-actin, the exposure time were 206 s and 32 s respectively. Densitometric scanning analyses for the protein expression of hERG1a (155 kDa and 135 kDa) and hERG1b (95 kDa and 80 kDa) are normalized against actin and relative to the untreated control. Densitometric data are presented as mean fold change ± SD of three independent experiments. *p < 0.05 compared with untreated control (one-way ANOVA followed with Dunnett’s post-hoc test). Refer to Supplementary Figs. 8 and 9 for full-length blots.

The effects of mitragynine on the mRNA expression levels of hERG1a and hERG1b were determined using RT-qPCR assay. As shown in Fig. 4A–D, mitragynine and arsenic trioxide did not affect the mRNA expression of both hERG1a and hERG1b at all concentrations (*p* > 0.05), suggesting that both compounds neither inhibit nor induce the mRNA expression of these isoforms. Arsenic trioxide was used as a control in this study as it is known to reduce the protein expression of hERG1a. However, its effects on mRNA expression were unknown. As expected, Western blot analysis showed that the expression of fg-hERG1a was significantly reduced (*p* = 0.0024) whereas the cg-hERG1a was significantly induced (*p* = 0.0058) by 1 µM arsenic trioxide (Fig. 5A,B). The results were in agreement with previously reported studies where arsenic trioxide was reported to reduce the expression of fg-hERG1a and increases the expression of cg-hERG1a due to disruption in hERG1a channel trafficking^39,40^. On the other hand, both fg- and cg-hERG1b protein expression were unaffected by arsenic trioxide at all concentration (*p* > 0.05).

Meanwhile, there was no clear pattern seen on the protein expression of fg-hERG1a upon treatment with 0.001–1 µM mitragynine (Fig. 5C,D). However, the expression of fg-hERG1a was significantly reduced by approximately 22.3% by 10 µM mitragynine (*p* = 0.0217). As for cg-hERG1a, the expression was unaffected by 0.001–1 µM mitragynine but was significantly increased by almost 50% when treated with 10 µM mitragynine (*p* = 0.0188). The results were similar to that observed for arsenic trioxide. On the other hand, there were no significant changes on the expression of fg- and cg-hERG1b treated by mitragynine at all concentration (*p* > 0.05) (Fig. 5C,D). The results suggested that mitragynine may be a hERG1a protein trafficking inhibitor like arsenic trioxide.

### Mitragynine reduces the CTCF/area of hERG1a expression

**Figure 6:**
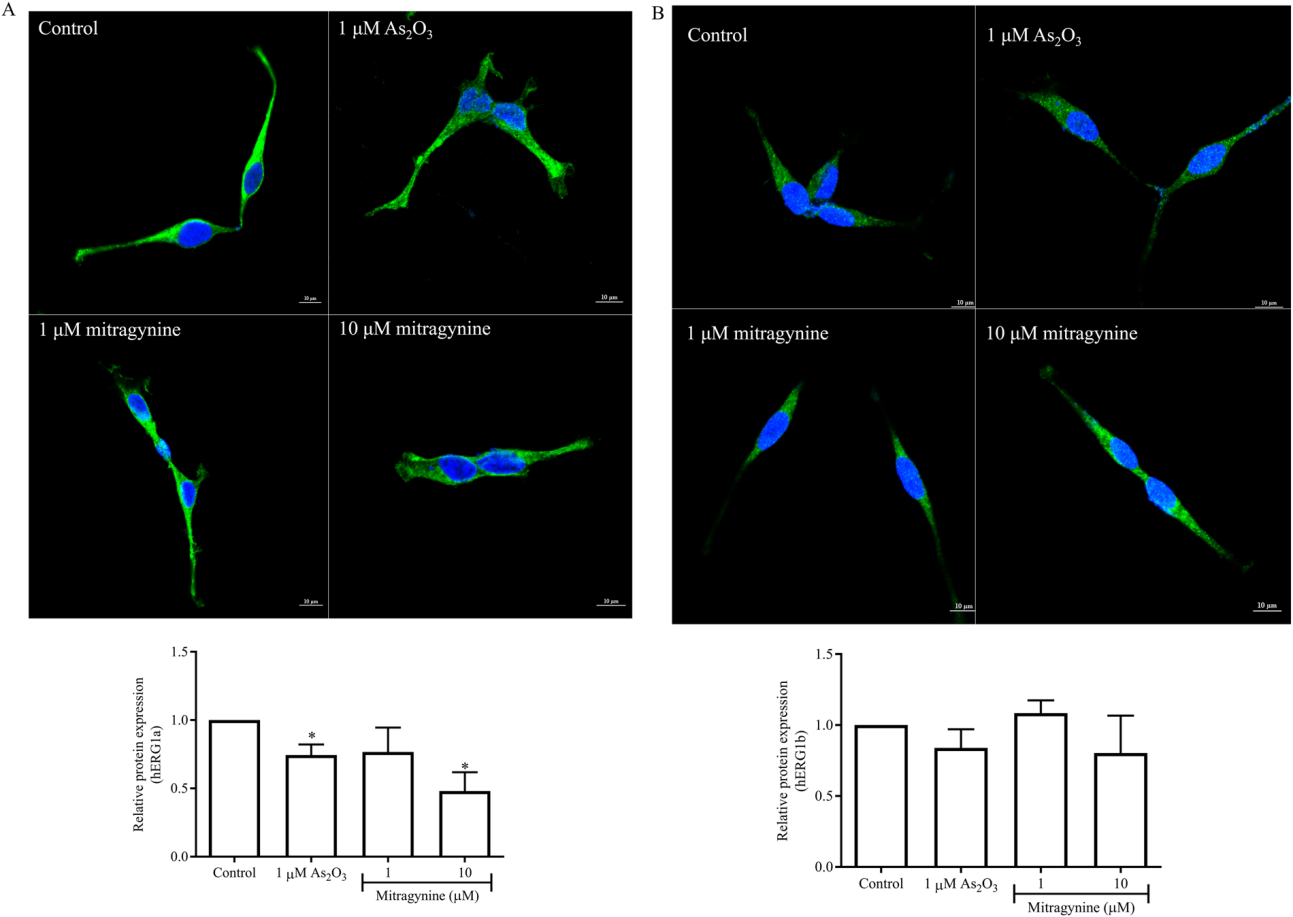
Immunofluorescence illustrating (**A**) hERG1a and (**B**) hERG1b surface protein expression in the HEK293-hERG1a/1b cells treated with 0.1% (v/v) DMSO, 1 μM arsenic trioxide, 1 μM mitragynine and 10 μM of mitragynine for 24 h. Semi-quantitation of fluorescence intensity relative to untreated control is illustrated in bar charts. Data are presented as mean corrected total cell fluorescence (CTCF) per area ± SD of three independent experiments. *p < 0.05 compared with untreated control (unpaired t-test).

Figure 6A,B shows the effects of arsenic trioxide and mitragynine on hERG1a and hERG1b surface expression. As expected, 1 µM arsenic trioxide significantly reduced the CTCF/area of hERG1a (*p* = 0.0047, Fig. 6A). On the other hand, cells treated with 10 µM of mitragynine for 24 h appeared to express hERG1a in a reduced manner as compared with control cells treated with 0.1% (v/v) DMSO (Fig. 6A). Semi-quantitative measurement of the fluorescence intensity showed a significant reduction to approximately half of the intensity when cells were treated with 10 µM mitragynine (*p* = 0.0028) as compared with control cells (Fig. 6A). On the other hand, the hERG1b fluorescence intensity was unaffected by both arsenic trioxide (*p* = 0.1016) and mitragynine (*p* = 0.1837 and 0.2667 for 1 μM and 10 μM respectively) (Fig. 6B). The immunofluorescence data concurred with the Western blot analysis results.

### Mitragynine significantly increases the relative expression of hERG1a/Hsp90 complexes

**Figure 7:**
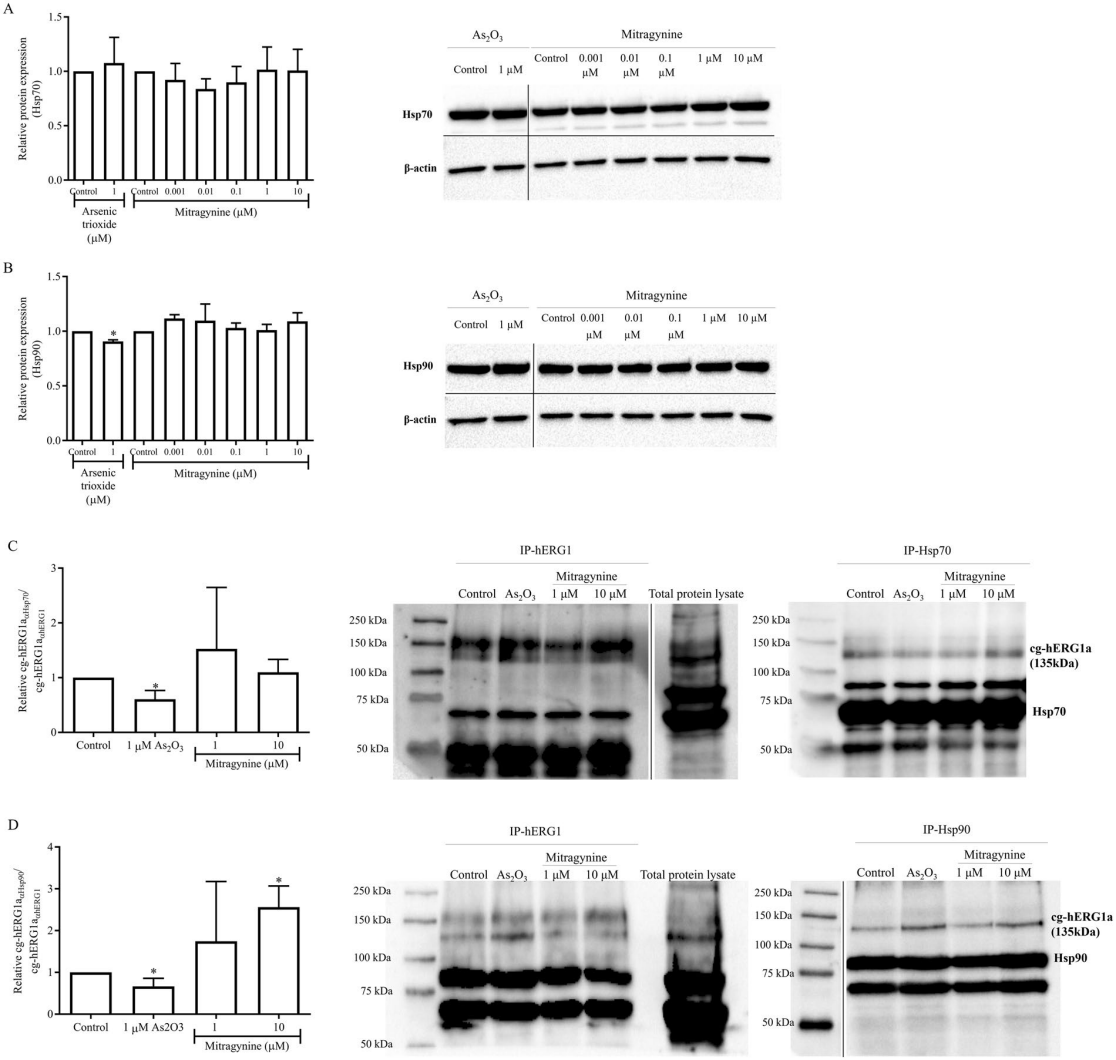
Densitometric scanning analysis and representative images of Western blot analysis for the protein expression of Hsp70 and Hsp90 following treatment with arsenic trioxide and mitragynine for 24 h. The effects of arsenic trioxide and mitragynine on the expression levels of endogenous (**A**) Hsp70 and (**B**) Hsp90. Densitometric scanning analysis and representative images for the protein expression of cg-hERG1a immunoprecipitated with anti-Hsp70 (IP-Hsp70) or anti-Hsp90 (IP-Hsp90) normalized against cg-hERG1a immunoprecipitated with anti-hERG1 (IP-hERG1) and relative to untreated control. The effects of arsenic trioxide and mitragynine on the interaction between cg-hERG1a (135 kDa) with (**C**) Hsp70 and (**D**) Hsp90. Electrophoresis was carried out at 150 V for 90 min and the protein bands were then transferred to a single PVDF membrane at 1.0 A and 25 V for 30 min. The protein of interest and the internal control of protein loading, β-actin (43 kDa), were separated by cutting the membrane at around 50 kDa using the molecular weight marker as reference. The separated membranes were then placed in different containers and incubated with respective antibodies. After immunoblotting, the separated membranes were placed together and visualized using ChemiDoc^™^ XRS Imaging System (Bio-Rad Laboratories Inc, USA). Hsp70 bands were detected with exposure time of 1.5 s, whereas β-actin bands were detected with exposure time of 10 s, meanwhile both Hsp90 & β-actin were detected with the exposure time of 7.6 s. For IP-hERG1, total protein lysates and proteins of interest were run on the same gel and blot with different exposure times for the proteins of interest (11 s) and total protein lysates (5 s). Data are presented as mean fold change ± SD of three independent experiments. *p < 0.05 compared with untreated control (unpaired t-test). Different blots were separated by black lines. Refer to Supplementary Figs. 10–13 for full-length blots.

The Western blot and immunofluorescence results suggested that trafficking of hERG1a from the endoplasmic reticulum (ER) to the Golgi may possibly be impaired in the presence of 10 µM mitragynine. Hence, the possibility of channel misfolding and interaction of hERG1a channels with cytosolic chaperones Hsp70 and Hsp90 were explored. Briefly, the effects of mitragynine and arsenic trioxide on the expression of endogenous Hsp70 and Hsp90 in HEK-hERG1a/1b cells were first determined (Fig. 7A,B). There was no significant changes on Hsp70 but the expression of Hsp90 was reduced slightly (approximately 10%) by 1 µM arsenic trioxide (*p* = 0.0005) (Fig. 7B). Meanwhile, neither Hsp70 nor Hsp90 levels were significantly altered (*p* > 0.05) after exposure to mitragynine for 24 h.

Subsequently, the effects of mitragynine on the association of hERG1a channels with Hsp70 and/or Hsp90 were investigated using co-immunoprecipitation methods. Figure 7C shows the effects of arsenic trioxide and mitragynine on the interaction between cg-hERG1a and Hsp70. For untreated control samples, fg- and cg-hERG1a as well as Hsp70 were co-immunoprecipitated with anti-hERG1 antibody (IP-hERG1). On the other hand, only cg-hERG1a and Hsp70 were co-immunoprecipitated with anti-Hsp70 antibody (IP-Hsp70), indicating that Hsp70 was in complex with the cg-hERG1a protein, but not the fg-hERG1a. For quantitative analysis, the expression of cg-hERG1a immunoprecipitated with anti-Hsp70 antibody was normalized against the expression of cg-hERG1a immunoprecipitated with anti-hERG1a. The relative expression of each treatment group was then compared to untreated control. In the presence of 1 µM arsenic trioxide, the formation of hERG1a/Hsp70 complexes was significantly inhibited by approximately 40% as compared with the untreated control (*p* = 0.0129). However, the association of hERG1a/Hsp70 was not significantly affected by both 1 µM (*p* = 0.4409) and 10 µM mitragynine (*p* = 0.4986) after 24 h of incubation.

Likewise, fg- and cg-hERG1a as well as Hsp90 were co-immunoprecipitated with anti-hERG1 antibody (IP-hERG1) in untreated control sample (Fig. 7D). Only cg-hERG1a and Hsp90 were co-immunoprecipitated with anti-Hsp90 antibody (IP-Hsp90), indicating that Hsp90 was in complex with the cg-hERG1a protein, but not the fg-hERG1a. Quantitative analysis showed that the formation of hERG1a/Hsp90 complexes was significantly inhibited by 1 µM arsenic trioxide by approximately 40% as compared with the untreated control (*p* = 0.0406). Interestingly, 10 µM mitragynine significantly increased the relative expression of hERG1a/Hsp90 complexes as compared with untreated control (*p* = 0.0101). Taken together, these data suggested that mitragynine-induced reduction of hERG1a protein is probably caused by an altered channel/Hsp90 interaction.

## Discussion

Kratom has been used for various medicinal and recreational purposes. Kratom is an euphoric agent and has a stimulant effect, hence, it is often misused for various reasons. Preparations containing this plant are not strictly regulated in certain countries and it can be easily obtained in various forms and doses via internet vendors. The products are often marketed as “legal highs” and “herbal highs”, giving the impression that the products are natural and safe to use^12^. To date, most of the poisoning and fatal cases reported involved the co-administration of kratom with other medications^41^. A number of mitragynine-related fatalities have been reported so far. For example, Holler and co-workers (2011) reported a drug toxicity death involving the use of mitragynine and propylhexedrine^42^. Kronstrand and co-workers (2011) published a fatality involving both mitragynine and O-desmethyltramadol (the active metabolite of tramadol)^11^. In addition, several other fatal cases related to kratom toxicity revealed mitragynine concentrations in the range of 0.23–1.06 mg/L in postmortem blood samples^12,13,41^. Recently, a possible case of kratom toxicity resulting in cardiac arrest and death was reported^43^. A 26-year-old man was admitted to the hospital with cardiorespiratory arrest after taken an unknown dose of kratom. Unfortunately, he deteriorated and died from cardiorespiratory failure and hypoxic brain damage 12 h after admission^43^.

Despite the growing number of poisoning and lethal cases related to kratom, the safety profile of the plant and its products remained poorly understood. Most of the *in vivo* toxicity studies of kratom extracts were conducted in animal models^15,44,45^. Although low doses do not pose any significant toxicity, the same cannot be said at higher doses (100–500 mg/kg). The toxicity affected body weight, behavior, internal organs and the blood of laboratory animals^8,45^. Mitragynine and kratom leaf extracts have also been shown to interact with the drug-metabolizing enzymes. The alkaloid extract has exhibited inhibitory effects on cytochrome P450 (CYP450) enzymes such as CYP3A4, CYP2D6 and CYP1A2^46^. In addition, mitragynine has shown potent inhibitory activity on CYP2D6 and an inductive effect on CYP1A2^47,48^. Thus, the potential of herb-drug interactions should also be considered when kratom is consumed concomitantly with other drugs which are substrates of the affected enzyme.

The effects of mitragynine and its derivatives on cardiac toxicity have been previously investigated in human induced pluripotent stem cell-derived cardiomyocytes (hiPSC-CMs). Mitragynine, paynantheine, speciogynine and speciociliatine suppressed the rapidly delayed rectifier potassium current (*I*_Kr_) in hiPSC-CMs by 67% - 84% and in a concentration-dependent manner. The IC_50_ values ranged from 0.91 to 2.47 µM. Mitragynine at 10 µM significantly prolonged the action potential duration (APD) at 50 and 90% repolarization respectively (APD50 and APD90)^49^. In addition, mitragynine was also found to inhibit the hERG1a and GIRK (G protein-coupled inward rectifier potassium) channels in other heterologous expression systems^50^. It was found to inhibit the hERG1a channel by interacting with the high-affinity binding sites, Y652 and F656 located in the S6 domain^50^. It is likely that homomeric hERG1a and heteromeric hERG1a/1b channels share the same drug binding sites, as both hERG1a and hERG1b isoform share the common sequence in the putative transmembrane region (S1–S6) including the pore loop between S5 and S6. However, drug sensitivity might vary between different hERG1 subunits due to their differences in gating kinetics^32,33^. For example, hERG1a channels were more rapidly inhibited by hERG1 blocker E-4031 as compared with hERG1a/1b channels^32^. In contrast, fluoxetine appeared to be a more potent blocker of hERG1a/1b than hERG1a channels^33^. Furthermore, multiple studies have confirmed the importance of hERG1a/1b in maintaining normal physiological function in the human heart^31,32,34,51^. Hence, apart from hERG1a channel, it is important to investigate inhibition potential of drugs on hERG1a/1b channels given the different gating properties and drug sensitivities of the isoforms. In heterologous expression system, hERG1a/1b gating properties appeared to better resemble those of native *I*_Kr_ current^29,30,32–34^.

In this study, the hERG1a/1b currents recorded showed typical voltage-dependent characteristics of hERG1 channel. To study the inhibitory effects of mitragynine on hERG1a/1b currents, a step pulse protocol was applied to evoke the tail current and the concentration-dependent inhibition was measured as the fractional reduction in peak tail current amplitude. Interestingly, mitragynine demonstrated a concentration-dependent inhibition on the hERG1a/1b current with an IC_50_ value of 332.70 nM (*h* = 0.61), which was rather similar to positive control drugs, for instance, propafenone and fluoxetine. The IC_50_ value was slightly lower than the previously determined IC_50_ values for mitragynine in hiPSC-CMs (0.91 μM), hERG1a-transfected HEK293 cells (1.62 μM) and hERG1a-injected *Xenopus* oocytes (1.03 μM)^49,50^. It is important to observe that the low IC_50_ values are also close to the mitragynine concentrations in postmortem blood samples, ranging from 0.57 μM to 2.66 μM^12,13,41^.

In addition to exerting an acute inhibitory effect on hERG1 channel, some drugs can alter hERG1 expression by affecting different steps within the trafficking pathway. In fact, more than 40% of hERG1 blockers presented combined acute channel blockade and trafficking defect^52,53^. Previous studies have suggested the potential of mitragynine in inhibiting hERG1a channel and *I*_kr_, but the effects of mitragynine on the channel trafficking were not fully elucidated^49,50^. Mitragynine was found to inhibit the protein expression of hERG1a, but the expression at the transcriptional level was not affected^50^. Similarly, in this study, mitragynine did not show any significant inhibitory or induction effects on the mRNA expression of hERG1a and hERG1b up to 10 μM, suggesting that mitragynine does not affect the transcriptional level of both isoforms. However, a reduction in fg-hERG1a and an increase in cg-hERG1a expression was observed after treatment with 10 μM mitragynine for 24 h. This result was further supported with the immunofluorescence experiments. The hERG1a expression in mitragynine-treated cells also showed a similar reduction in the fluorescence intensity. Numerous studies have shown that misfolding of hERG1a channels is the leading cause of defects in channel trafficking. The cytosolic chaperones Hsp70 and Hsp90 are two important chaperones that assist in channel folding and thus required for the biochemical maturation of hERG1 channel^40,54^. One of the possible explanations for impaired forward trafficking is the failure of the cg-hERG1a channel to interact with cytoplasmic chaperones such as Hsp70 and Hsp90, leading to channel misfolding at the cellular level and the inability to traffic from the ER to the plasma membrane^55^. In this study, arsenic trioxide inhibited the expression of Hsp90 by approximately 10% but did not significantly inhibit the expression of Hsp70. On the other hand, mitragynine did not exert any effect on both Hsp70 and Hsp90 expression. The level of hERG1a protein interacting with Hsp70 and/or Hsp90 was significantly decreased by long-term exposure of 1 μM arsenic trioxide. This was expected since arsenic trioxide is known to affect channel forward trafficking from the ER through the inhibition of hERG1a-Hsp70 and hERG1a-Hsp90 complexes^39,40^. Surprisingly, mitragynine did not inhibit the hERG1a-Hsp70 and hERG1a-Hsp90 interactions like arsenic trioxide. On the contrary, 10 μM mitragynine significantly up-regulated the levels of hERG1a interacting with Hsp90 by approximately 2.5-fold as compared with the untreated control. The increase in interaction between hERG1a and Hsp90 is expected to promote proper hERG1a channel folding and subsequently increases the fg-hERG1a protein expression at plasma membrane. However, this was not observed.

Another possible explanation is that mitragynine may induce hERG1a channel misfolding which leads to accumulation of hERG1a-Hsp90 complexes and ER retention. In a study conducted by Ficker and co-workers (2003), the interactions of the trafficking-deficient LQT2 mutants, hERG1 R752W and hERG1 G601S, with Hsp90 and Hsp70 were found to increase as both mutants remained tightly associated with Hsp90 and Hsp70 in the ER, which support the observation that both proteins are crucial for the retention of trafficking deficient LQT2 mutants^56^. Hence, it is hypothesized that mitragynine may prevent proper hERG1a channel folding leading to failure in hERG1a channel trafficking to the plasma membrane. In addition, it is also possible that the accumulation of hERG1a-Hsp90 complexes may cause hERG1a ubiquitination and degradation via lysosomal or proteasomal pathway upon overnight exposure to mitragynine. This mechanism may be similar to that reported for desipramine, an antidepressant agent. It was found to inhibit hERG1a forward trafficking by inducing aggregation of near-native hERG1a channels in the ER which were subsequently ubiquitinated and degraded via lysosomal pathway^57^. Nevertheless, whether mitragynine triggers the unfolded protein response (UPR) and endoplasmic-reticulum-associated protein degradation (ERAD) system is not known at this point of time. The UPR, which is indicative of an ER-stress response, is a process that prevents the accumulation of unfolded proteins in the ER^40,58^. More in-depth study on possible mitragynine-induced ER retention is certainly warranted.

Although patch clamp experiments do offer a direct information of hERG1 channel function, data measured in heterologous expression systems may not fully represent channel function in normal physiological condition^59^. This is mainly because the electrophysiology of cardiomyocytes is regulated by multiple ion channels that work simultaneously^60^. For instance, verapamil inhibits hERG1 channels at clinically relevant concentrations, but it has no known risk for TdP due to its concomitant blockade of the depolarizing inward calcium current (*I*_Ca,L_, through blockade of the calcium channel subunit Cav1.2), which alleviates the effects of reduced *I*_Kr_ outward current^20,61^. In recent years, the CiPA (Comprehensive *in vitro* Proarrhythmia Assay) initiative was given much attention to overcome these limitations by assessing drug electrophysiology on multiple cardiac ion channels *in vitro* and subsequently integrating these data into computational models of adult human ventricular electrical activity^62^. Computational action potential models which include hERG1a/1b isoforms were also developed^32,63^. Hence, integration of the mitragynine inhibition data collected from patch clamp experiment with these models may allow a more comprehensive evaluation of cardiac toxicity of mitragynine.

In conclusion, this study provides insight into the molecular mechanism of mitragynine inhibition on the hERG1a/1b channel. At lower concentration, mitragynine directly inhibited the hERG1a/1b current with an IC_50_ of 332.70 nM. At higher concentration (10 μM), hERG1a protein expression was significantly reduced by mitragynine but not at the transcriptional level suggesting that mitragynine indirectly inhibits hERG1a surface expression possibly through impaired trafficking. Significant up-regulation of hERG1a-Hsp90 complexes may possibly indicate a different trafficking inhibition pathway by mitragynine as compared with arsenic trioxide. This study suggests a significant *in vitro* cardiac toxicity risk of mitragynine and provides useful information for the use and regulation of mitragynine or kratom as an herbal product.

## Materials and Methods

### Isolation and characterization of mitragynine

Mitragynine was isolated and characterized as previously described^49^. The fresh leaves of *M. speciosa* Korth. (Rubiaceae) were collected from the state of Perlis, Malaysia with permission from the Ministry of Health Malaysia and the Narcotics Crime Investigation Department, Royal Malaysia Police (PDRM). The authentication of plant material was carried by the Forest Research Institute Malaysia (FRIM). Briefly, the fresh leaves were first dried using a drying oven at 50 °C for a week and subsequently processed into powdered dried material. It was then extracted with methanol for 72 h using soxhlet apparatus before filtered and evaporated under reduced pressure at 40–45 °C to obtain methanol extract. The extract was subsequently dissolved in 10% (v/v) acetic acid and left to stand for 24 h which was then filtered to yield the acidic filtrate. The filtrate was then washed with n-hexane and 25% (v/v) ammonia solution and finally extracted using portions of chloroform. The chloroform extract was then mixed with anhydrous sodium sulfate and evaporated to yield crude alkaloid extract. The crude alkaloid was fractionated using SiO_2_ column chromatography with n-hexane and ethyl acetate eluent system and mitragynine eluted accordingly. The eluted fraction was also subjected to preparative- TLC to increase the purity of the compound. Subsequently, the fraction was evaporated to dryness and recrystallized with n-hexane and diethyl ether to produce an approximately 30% (w/w) of mitragynine. The structure and identity of the compounds were confirmed using 1H-NMR and 13C-NMR analysis as shown in Supplementary Fig. 7.

### Cell culture and medium

Human embryonic kidney (HEK) 293 cells (ATCC^®^ CRL-1573^™^, USA) were maintained in Dulbecco’s Modified Eagle medium (DMEM) (Gibco, USA) and supplemented with 10% (v/v) fetal bovine serum (FBS) (Gibco, USA), 1 mM sodium pyruvate (Sigma-Aldrich, USA) and 1x MEM non-essential amino acid solution (Sigma-Aldrich, USA). Cultures were incubated at 37 °C in humidified incubator supplemented with 5% (v/v) CO_2_.

### Molecular cloning and the construction of stable hERG1a/1b-transfected HEK293 cells

The stable HEK293-hERG1a cell line was previously established in the laboratory by transfecting HEK293 cells with pCMV6-Neo-hERG1a plasmid (OriGene, USA)^50^. The stable HEK293- hERG1a cells were maintained in DMEM complete medium supplemented with 500 µg/ml G418 sulfate (Geneticin^®^) (Gibco, USA). To generate hERG1b-containing expression vector [pcDNA^™^3.1/Zeo(+)], the hERG1b cDNA (3.3 kb) was first released from pCMV6-XL4-hERG1b (OriGene, USA) using restriction enzyme (RE) NotI-HF^™^ (New England BioLabs, Inc., USA) before ligation with the linearized vector. Purified recombinant plasmid was subjected to DNA sequencing and analyzed using Nucleotide Basic Local Alignment Searching Tool (BLAST) from National Center for Biotechnology Information (NCBI) (http://blast.ncbi.nlm.nih.gov) to ensure the authenticity of the sequence. To generate hERG1a/1b recombinant cell line, an approximately 100 µl of HEK293-hERG1a cell suspension at the concentration of 1 × 10^6^ cells/ml were transfected with 2.5 µg purified pcDNA™3.1/Zeo(+)-hERG1b plasmid via electroporation using Gene Pulser Xcell™ Electroporation system (Bio-Rad Laboratories, USA). Electroporation was carried out at 110 V for 25 ms. The cells were then selected in complete medium supplemented with optimized concentrations of G418 sulfate (500 µg/ml) and Zeocin™ (300 µg/ml) (Gibco, USA). Subsequently, the cells were cultured in selection medium for up to 30 days, and single colonies were manually picked and propagated in fresh selection medium for several weeks. To confirm the presence of hERG1a/1b expression, clonal cell lines were screened using RT-qPCR and Western blot analyses. Patch clamp electrophysiology system was used to characterize and measure the hERG1a/1b current. For these studies, only cells from low passage number (less than 20 passages) were used, and the hERG1a/1b current was verified for every new batch of cells.

### Cell viability and proliferation assay

Trypan blue exclusion test was carried out to determine the effect of transfection on HEK293-hERG1a/1b cell viability. Briefly, cell suspension was mixed with 0.4% (v/v) trypan blue solution in a ratio of 1:1 and the viability of the cells were analyzed using Countess^®^ II FL automated cell counter (Invitrogen, USA). For cell proliferation assay, cells were first seeded at a density of 5000 cells/well onto two 96-well plates (T_0_ and T_1_) and were incubated at 37 °C for 24 h in a humidified incubator supplemented with 5% (v/v) CO_2_. Subsequently, cells in T_0_ plate were subjected to cell proliferation assay as described previously^64^. Meanwhile, cells in T_1_ plate were cultured in reduced serum medium (0.5% v/v FBS) for 4 h and treated with various concentrations of mitragynine. After 72 h, cell proliferation was determined using CellTiter 96^®^ AQ_ueous_ One Solution Cell Proliferation Assay (Promega, USA) following manufacturer’s protocol. Cell proliferation results were expressed as percentage growth (PG)^65^. The response parameters GI_50_ (half maximal growth inhibitory concentration), TGI (total growth inhibition) and LC_50_ (half maximal lethal concentration) values were derived from a non-linear regression model (curvefit) based on dose response-inhibition curve and computed using GraphPad Prism^®^ 5.01 (GraphPad Software, USA). The GI_50_, TGI and LC_50_ are interpolated values representing the concentrations at which the PG is +50, 0 and −50 respectively. Data are presented as mean PG for three independent experiments.

### Electrophysiology using Port-a-Patch system

The extracellular solution for current recordings contained 138 mM NaCl, 6 mM KCl, 1 mM MgCl_2_, 2 mM CaCl_2_, 5 mM D-glucose monohydrate and 10 mM HEPES (titrated to pH 7.4 ± 0.2 with 1 M NaOH; osmolarity 298 mOsmol ± 5 mOsmol). The intracellular solution contained 50 mM KCl, 10 mM NaCl, 60 mM potassium fluoride, 20 mM EGTA, 10 mM HEPES (titrated to pH 7.4 ± 0.2 with 1 M KOH; osmolarity 285 mOsmol ± 5 mOsmol). Seal enhancer solution consisted of 80 mM NaCl, 3 mM KCl, 10 mM MgCl_2_, 35 mM CaCl_2_, 10 mM HEPES-sodium salt (titrated to pH 7.4 ± 0.2 with 1 M HCl; osmolarity 298 mOsmol ± 5 mOsmol). All working solutions were sterile-filtered, stored at −20 °C and thawed to room temperature before use.

Prior to electrophysiology study, the HEK293-hERG1a/1b cells were first grown in complete medium without selection antibiotics for 6 days. Approximately 5.0 × 10^5^ cells were then plated onto an uncoated 96-mm cell culture dish for a maximum of 3 days. Cells were maintained in reduced-serum medium containing 2.5% (v/v) FBS the night before electrophysiology studies and subsequently harvested by incubating in 1 ml TrypLE^™^ Express (Gibco, USA) for about 30 s at 37 °C. The dissociation enzyme was immediately diluted with 5 ml DMEM and the cells were gently pipetted several times to obtain single cells. The cell suspension was then transferred into a 15-ml polypropylene tube and centrifuged at 100 × *g* for 3 min. Subsequently, the cell pellet was resuspended in extracellular solution to obtain a final density of 2 × 10^6^–3 × 10^6^ cells/ml and incubated on ice for 20 min to allow cell recovery. The electrophysiology studies were performed using NPC^®^-1 chips (Nanion Technologies, Germany) and Port-a-Patch system (Nanion Technologies, Germany) following manufacturer’s instructions, within the next 2 h. For characterization and determination of the hERG1a/1b current inhibition, a series of voltage protocols were applied as published previously^50^.

### Voltage command protocols for hERG1a/1b current in hERG1a/1b-transfected HEK293 cells

The hERG1a/1b current was recorded in cells achieving whole-cell configuration. Briefly, a single cell was first held at −80 mV, the theoretical cardiac resting membrane potential, on the aperture of the NPC-1 chip (Nanion Technologies, Germany). To characterize the I-V relationship, the cell was depolarized to test potentials ranging from −60 mV to +50 mV for 4 s in 20-mV increment, followed by a 5-s step to −50 mV before returning to −80mV. The I-V plot was fitted to Boltzmann function: I/I_max_ = |1 + *exp*[(V_1/2_ − V)/*k*]|^−1^, using GraphPad Prism^®^ 5.01 (GraphPad, USA), where I/I_max_ is the normalized tail current, V_1/2_ is the voltage of half maximal activation, V is the membrane potential, and *k* is the slope factor

The fully activated I-V relationship was determined using depolarization step to +60 mV for 1 s, followed by repolarization potentials ranging from −120 mV to +40 mV for 1 s in 20-mV increment. The peak tail currents (I_tail_) recorded upon repolarization were used to compute the fully-activated I-V curve. On the other hand, the rate of inactivation was evaluated by employing a three-pulse protocol^32,33,66^. The hERG1a/1b current was first fully activated and inactivated by a depolarizing step to +60 mV for 200 ms, followed by a brief hyperpolarizing step to −100 mV. The hyperpolarizing step was set for 2 ms to prevent significant deactivation from occurring. The membrane potential was then stepped to a series of test pulses between −20 mV and +60 mV, allowing the rate of inactivation to be measured as a function of voltage. The time constants for onset of inactivation were estimated by fitting the decay of the currents in the third pulse to a single exponential function and plotted as a function of test potential.

A two-pulse protocol was used to measure the time constant for recovery from inactivation. The measurement was performed by activating and inactivating the hERG1a/1b current at +60 mV for 200 ms, followed by repolarization to potentials ranging from −20 to −100 mV to record a tail current^33,66^. The time constants for recovery from inactivation were measured as the single exponential fit to the tail current rising phase (>−60 mV) or as the fast time constant (τ_fast_) of a double exponential fit (≤−60 mV) to the tail current^33^. Exponential fits were analyzed using GraphPad Prism^®^ 5.01 software (GraphPad, USA).

To determine the pharmacological effects of the HEK293-hERG1a/1b cells, the cells were perfused with test compound solutions using an external perfusion system. Before application of the test compounds, the hERG1a/1b current amplitude was required to be stable for at least 6 continuous readings. The mean of six successive I_tail_ was recorded for each test concentration. Four independent experiments were conducted to derive the dose-response curve. To determine compound potency, the tail current amplitude for each concentration was normalized to control, i.e. 0.1% (v/v) DMSO. IC_50_ values were derived by fitting the normalized dose-response data to the Hill equation, Y = bottom + (Top - bottom)/[1 + 10^(logIC50-X)*h^], using GraphPad Prism^®^ 5.01 (GraphPad, USA), where Y is the normalized relative current (I/I_baseline_), X is the concentration, IC_50_ is the half maximal block concentration, and *h* is the Hill coefficient^67,68^.

### Quantitative RT-PCR (RT-qPCR)

Briefly, total cellular RNA was isolated from treated cells using RNeasy^®^ Plus Mini kit (Qiagen, Germany) following manufacturer’s instruction. The optimized RT-qPCR assay was performed using CFX96^™^ real-time PCR system (Bio-Rad Laboratories Inc., USA) with the following cycle parameters: 10 min at 50 °C for reverse transcription, 1 min at 95 °C for activation of Taq polymerase, followed by 40 cycles of 10 s at 95 °C for denaturation and 30 s at 53.9 °C for annealing and data acquisition. Specifically, the assay was performed using 100 ng of total RNA and iTaq^™^ Universal SYBR^®^ Green One-Step Kit (Bio-Rad Laboratories Inc., USA) in 10 µl of reaction volume. A melt curve analysis was computed at the end of the qPCR cycles to determine the specificity of the primers. The results were analyzed using CFX Manager software version 3.1 (Bio-Rad Laboratories Inc., USA). The mRNA expressions of hERG1a/1b were presented as fold changes normalized to endogenous reference genes, ACTB and GAPDH, and relative to the control gene^69–71^. Each experiment was conducted in triplicate and data are presented as mean fold changes of three independent experiments.

### Western blot analysis

Total protein was isolated using RIPA lysis buffer system (Santa Cruz Biotechnology, Inc., USA). Equal amounts of protein samples were loaded onto 7.5% (v/v) polyacrylamide gels and were subsequently transferred to Immobilon^®^-P PVDF membrane (Millipore, USA) using Trans-Blot^®^ Turbo^™^ Blotting System (Bio-Rad Laboratories, USA). Blots were blocked with 3% (w/v) skim milk for 1 h and probed with optimized primary antibodies against hERG1a (1:5000), hERG1b (1:1250), Hsp70 (1:10, 000), Hsp90 (1:5000) and β-actin (1:5000) overnight at 4 °C. Finally, the blots were then incubated in horseradish peroxidase (HRP) conjugated secondary antibody (1:1000) for 2.5 h followed by Clarity^™^ Western ECL substrate (Bio-Rad Laboratories, USA). The antibodies used for Western blotting were rabbit hERG1a polyclonal antibody (Enzo Life Sciences, USA), rabbit hERG1b polyclonal antibody (Enzo Life Sciences, USA), rabbit Hsc70/Hsp70 polyclonal antibody (Enzo Life Sciences, USA), rabbit Hsp90 polyclonal antibody (Enzo Life Sciences, USA), rabbit β-actin antibody (Cell Signaling, USA) and anti-rabbit IgG HRP-linked antibody (Cell Signaling, USA). Chemiluminescence signal was detected using ChemiDoc^™^ XRS Imaging System (Bio-Rad Laboratories, USA). Band densities were scanned and quantified using Image Lab Software (Bio-Rad Laboratories, USA). The signal for each protein band was normalized to the reference protein, β-actin. Experiments were conducted in duplicate and represented as mean fold changes of three independent experiments.

### Immunofluorescence

Briefly, cells were first seeded onto 24 mm × 24 mm pre-coated glass cover slips (Leica Microsystems, Germany) in a 6 well plate containing 10% (v/v) FBS-complete DMEM medium. The sterile glass cover slips (Leica Microsystems, Germany) were coated with HistoGrip (Invitrogen, USA) before use. Cells were first cultured in fresh medium containing 0.5% (v/v) FBS for 4 h before replenishing with medium containing arsenic trioxide (positive control) or mitragynine for 24 h. Medium containing 0.1% (v/v) DMSO was used as control. After incubation, the monolayer cells were fixed with 4% (w/v) paraformaldehyde and incubated for 15 min at room temperature, followed by rinsing with PBST solution and incubating with 0.1% (v/v) Triton X-100 solution for 2 min. Subsequently blocking was performed with 3% (w/v) BSA for 1 h and followed by incubation with primary antibodies against hERG1a (1:1000) and hERG1b (1:125) at 4 °C overnight. Immunolabelling was carried out using anti-rabbit IgG F(ab’)_2_ Alexa Fluor^®^ 488 molecular probes (Cell Signaling, USA) diluted at 1:250 in 3% (w/v) BSA at room temperature. Finally, the cover slips were mounted using Fluoroshield^™^ with DAPI (Sigma-Aldrich, USA) and viewed under 600x magnification using Nikon C2 confocal microscope (Nikon, USA). The excitation/emission wavelengths for Alexa Fluor 488 and DAPI were 488/512 nm and 405/488 nm respectively. The laser imaging settings such as laser power, gain and amplification factor were set to be similar for all samples. Briefly, 15 spots (images) per slide consisting 1–3 cells for each image were chosen. For each test sample, at least 20 cells were randomly selected from the 15 spots for quantitation. The green fluorescence intensity was quantified using ImageJ software (http://imagej.nih.gov/ij/)^72,73^. Intensity was represented as the “corrected total cell fluorescence” (CTCF) per area using the equation “CTCF/area = [Integrated density - (Area of selected cell × Mean fluorescence of background readings)]/Area of selected cell”. Relative protein expression of hERG1a and hERG1b was determined by dividing the mean CTCF/area of treated cells to that of the untreated control. The experiment was carried out in single replicate for three independent experiments.

### Co-immunoprecipitation

The co-immunoprecipitation method was adapted and modified slightly from the previously described methodologies^40,52^. The treated recombinant cells were incubated for 15 min at room temperature with PBS containing cross-linker 1.5 mM dithiobis (succinimidyl propionate) (DSP, Pierce USA). The cross-linker was quenched by addition of 10 mM Tris (pH 7.5) and incubated for 10 min at room temperature. Cells were detached in 10 ml ice-cold PBS and centrifuged at 300 *x g* for 5 min at 4 °C. The pellet was then resuspended in 300 µl ice-cold complete diluted RIPA lysis buffer containing 0.1% (v/v) NP40, 50 mM Tris pH 7.5, 150 mM NaCl, 1x PMSF solution, 1x sodium orthovanadate solution and 1x protease inhibitor cocktail solution. Subsequently, the cell suspension was incubated at 4 °C for 30 min with constant agitation and finally centrifuged at 14, 000 rpm for 10 min at 4 °C. The supernatant was saved for total protein quantitation and subsequently for co-immunoprecipitation experiments.

Briefly, 500 µg of isolated total protein was mixed with 2 µg of hERG1 rabbit polyclonal IgG (Santa Cruz Biotechnology Inc., USA), mouse Hsp70/Hsc70 monoclonal antibody (Santa Cruz Biotechnology Inc., USA) or mouse Hsp90α/β monoclonal antibody (Santa-Cruz Biotechnology Inc., USA) respectively and incubated on ice on a rotary shaker overnight. Subsequently, 30 µl of protein A/G PLUS-agarose immunoprecipitation reagent (Santa Cruz Biotechnology Inc., USA) was added into the mixture, and incubated on ice on a shaker for 8 h and subsequently stored in 4 °C overnight. The mixture was centrifuged at 1500 rpm for 5 min at 4 °C, and the pellet was washed with ice-cold PBST, each time repeating the centrifugation process. Finally, the pellet was resuspended in 25 µl 1x Laemmli sample buffer (Bio-Rad Laboratories Inc., USA) with 2.5% (v/v) β-mercaptoethanol, and then heated at 70 °C for 2 h to reverse cross-linking and to release the immunoprecipitated protein from A/G PLUS-agarose beads. The samples were centrifuged at 1500 rpm for 5 min at 4 °C to pellet the agarose beads and 20 µl of the supernatant was used for subsequent SDS-PAGE and immunoblotting analysis as described previously.

To analyze the immunoprecipitated proteins, the membrane was probed with rabbit anti-potassium channel Kv11.1 extracellular antibody (1:200) (Sigma-Aldrich, USA), mouse Hsp70/Hsc70 monoclonal antibody (1:10, 000) and mouse Hsp90α/β monoclonal antibody (1:10, 000) in the same container. Protein A (HRP conjugate) (Cell Signaling, USA) was used as secondary antibody to detect both rabbit immunoglobulin (IgG) and mouse IgG2a or IgG2b^74^. The experiment was carried out in single replicate for three independent experiments. To quantify hERG1/chaperone interactions, image densities of the core-glycosylated (cg) hERG1 protein found in immunoprecipitations with anti-hERG1 antibody were used as a measure of total hERG1a protein present. Image densities of hERG1 protein bands isolated with anti-chaperone antibodies (anti-HSP70 and anti-HSP90) were normalized to image densities of hERG1 protein bands isolated with anti-hERG1 antibody to compute association quotients^39,58^.

### Data analysis

One-way ANOVA followed by Dunnett’s post-hoc test or unpaired Student t-test using GraphPad Prism^®^ 5.01 (GraphPad Software, USA) were used to test the significance of experiments. Results are displayed as mean ± SD and are considered statistically significant when *p* values < 0.05.

## Supplementary information

Supplementary information

## Data Availability

Experimental data will be made available upon request.
