# Supplementary material for: Mitragynine, an euphoric compound inhibits hERG1a/1b channel current and upregulates the complexation of hERG1a-Hsp90 in HEK293-hERG1a/1b cells
Source: Sci Rep. 2019 Dec 24;9:19757. doi: 10.1038/s41598-019-56106-6 (PMC6930223; doi:10.1038/s41598-019-56106-6)
Supplement: Supplementary file 1 — Supplementary information [file 41598_2019_56106_MOESM1_ESM.pdf]

Supplementary Figures

Mitragynine, an euphoric compound inhibits hERG1a/1b channel current and upregulates the complexation of hERG1a-Hsp90 in HEK293-hERG1a/1b cells

Yea Lu Tay[1], Azimah Amanah[1], Mohd Ilham Adenan[2], Habibah Abdul Wahab[3], Mei Lan Tan[1,3,4*]

[1]Malaysian Institute of Pharmaceuticals & Nutraceuticals, NIBM, Ministry of Energy, Science, Technology, Environment and Climate Change (MESTECC), Pulau Pinang, 11700 Malaysia

[2]Atta-ur-Rahman Institute for Natural Product Discovery, Universiti Teknologi MARA (UiTM), Selangor Darul Ehsan, 42300 Malaysia

[3]School of Pharmaceutical Sciences, Universiti Sains Malaysia, Pulau Pinang, 11700 Malaysia

[4]Advanced Medical and Dental Institute, Universiti Sains Malaysia, SAINS@BERTAM, Kepala Batas, Pulau Pinang, 13200 Malaysia.

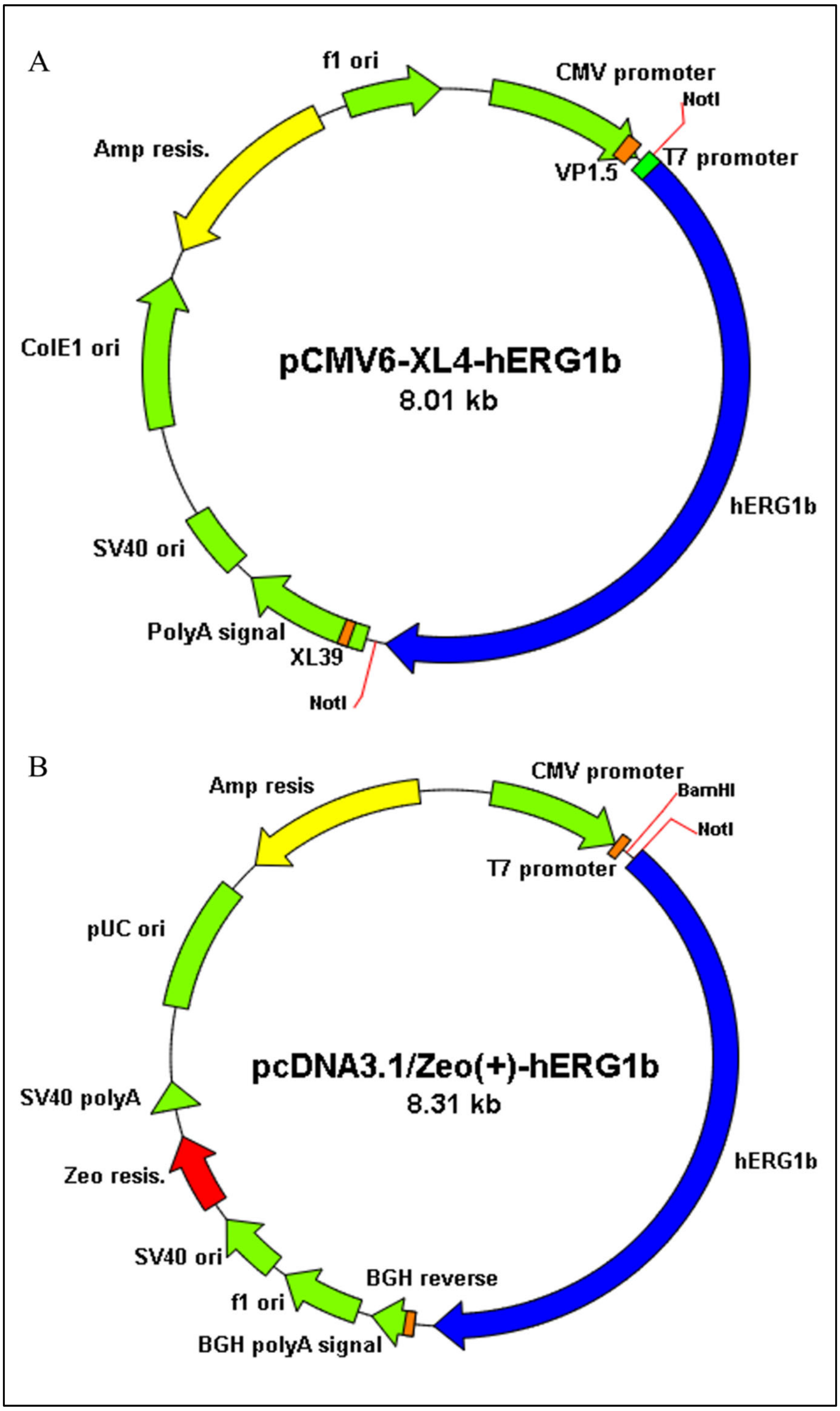

Supp Fig. 1 Plasmid map of (A) pCMV6-XL4-hERG1b and (B) pCDNA™3.1/Zeo(+)-hERG1b (adapted from OriGene Technologies, USA and Invitrogen, USA).

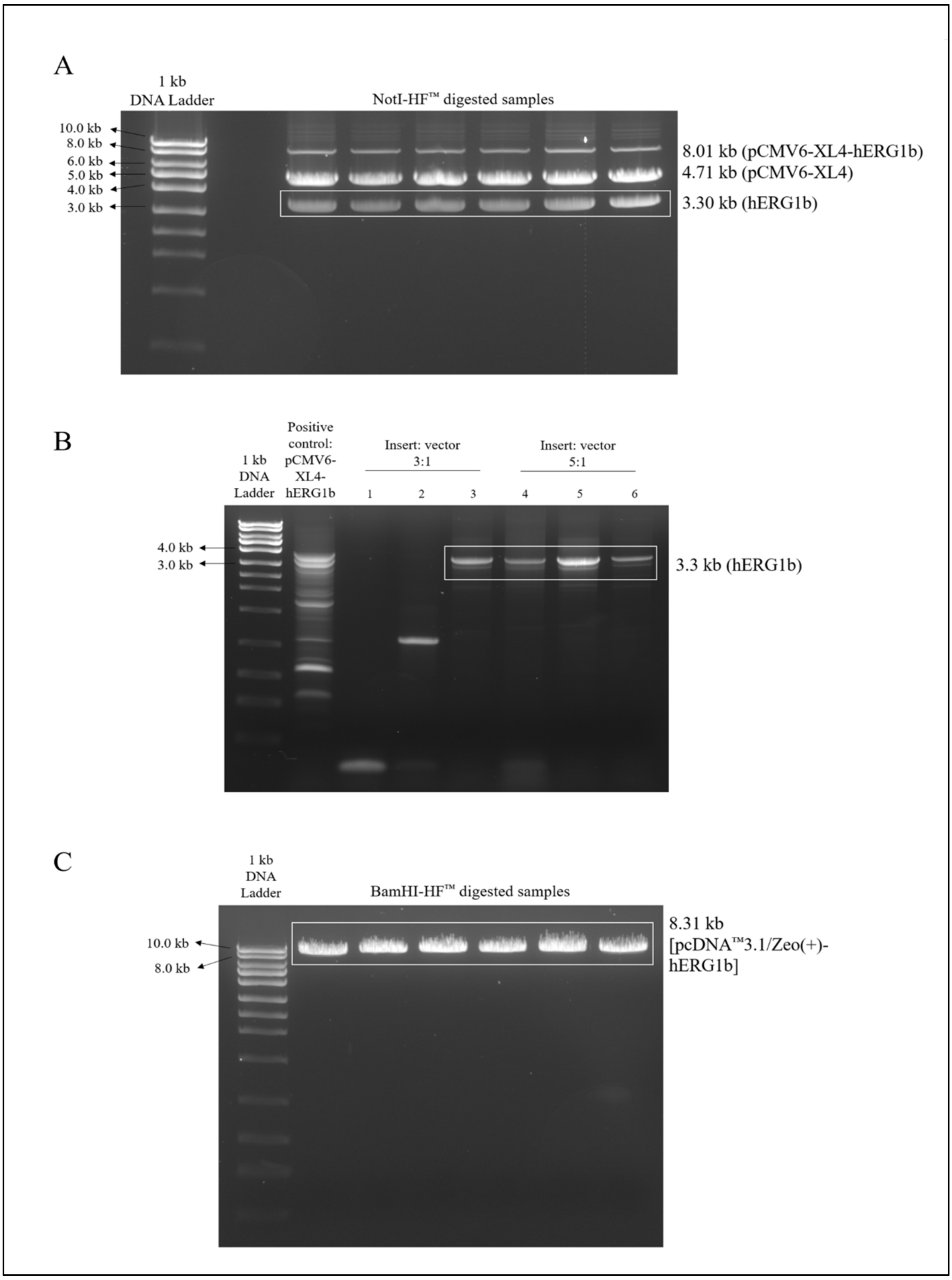

Supp Fig. 2 Gel electrophoresis images (A) NotI-HF™ restricted pCMV6-XL4-hERG1b samples, where 8.01 kb refers to the pCMV6-XL4-hERG1b recombinant plasmid, 4.71 kb refers to the fragment of plasmid, and 3.30 kb refers to the hERG1b cDNA insert. (B) Colony PCR products of six colonies of transformed competent cells with recombinant pcDNA™3.1/Zeo(+)-hERG1b in insert: vector ratios of 3:1 (Lane 1-3) and 5:1 (Lane 4-6), where ratio of 5:1 was the optimized proportion for successful ligation of hERG1b into pcDNA™3.1/Zeo(+)-hERG1b. (C) BamHI-HF™ restricted pcDNA™3.1/Zeo(+)-hERG1b samples, where 8.31 kb refers to the size of the linearized recombinant plasmid.

Homo sapiens potassium voltage-gated channel, subfamily H (eag-related), member 2 (KCNH2), transcript variant 3, mRNA

Sequence ID: ref|NM_172057.2| **Length:** 3212 **Number of Matches:** 1

Range 1: 351 to 770 GenBank Graphics

Score	Expect	Identities	Gaps	Strand
776 bits(420)	0.0	420/420(100%)	0/420(0%)	Plus/Plus

```
Query  1    AGCAGGACAGGGGCTCTGCGGCCCAGGGCCCAGAAAGGCCGGGTGAGGCGGGCCGTGCGC  60
            ||||||||||||||||||||||||||||||||||||||||||||||||||||||||||||
Sbjct  351  AGCAGGACAGGGGCTCTGCGGCCCAGGGCCCAGAAAGGCCGGGTGAGGCGGGCCGTGCGC  410

Query  61   ATCTCCAGCCTCGTGGCCCAGGAGGTCCTGTCCCTGGGCGCCGACGTGCTGCCTGAGTAC  120
            ||||||||||||||||||||||||||||||||||||||||||||||||||||||||||||
Sbjct  411  ATCTCCAGCCTCGTGGCCCAGGAGGTCCTGTCCCTGGGCGCCGACGTGCTGCCTGAGTAC  470

Query  121  AAGCTGCAGGCACCGCGCATCCACCGCTGGACCATCCTGCATTACAGCCCCTTCAAGGCC  180
            ||||||||||||||||||||||||||||||||||||||||||||||||||||||||||||
Sbjct  471  AAGCTGCAGGCACCGCGCATCCACCGCTGGACCATCCTGCATTACAGCCCCTTCAAGGCC  530

Query  181  GTGTGGGACTGGCTCATCCTGCTGCTGGTCATCTACACGGCTGTCTTCACACCCTACTCG  240
            ||||||||||||||||||||||||||||||||||||||||||||||||||||||||||||
Sbjct  531  GTGTGGGACTGGCTCATCCTGCTGCTGGTCATCTACACGGCTGTCTTCACACCCTACTCG  590

Query  241  GCTGCCTTCCTGCTGAAGGAGACGGAAGAAGGCCCGCCTGCTACCGAGTGTGGCTACGCC  300
            ||||||||||||||||||||||||||||||||||||||||||||||||||||||||||||
Sbjct  591  GCTGCCTTCCTGCTGAAGGAGACGGAAGAAGGCCCGCCTGCTACCGAGTGTGGCTACGCC  650

Query  301  TGCCAGCCGCTGGCTGTGGTGGACCTCATCGTGGACATCATGTTCATTGTGGACATCCTC  360
            ||||||||||||||||||||||||||||||||||||||||||||||||||||||||||||
Sbjct  651  TGCCAGCCGCTGGCTGTGGTGGACCTCATCGTGGACATCATGTTCATTGTGGACATCCTC  710

Query  361  ATCAACTTCCGCACCACCTACGTCAATGCCAACGAGGAGGTGGTCAGCCACCCCGGCCGC  420
            ||||||||||||||||||||||||||||||||||||||||||||||||||||||||||||
Sbjct  711  ATCAACTTCCGCACCACCTACGTCAATGCCAACGAGGAGGTGGTCAGCCACCCCGGCCGC  770
```

Supp Fig. 3 Sequence alignment and comparison between the sequences of the cDNA insert (Query) against hERG1b sequence [NM_172057.2; Homo sapiens potassium voltage-gated channel, subfamily H (eag-related), member 2 (KCNH2), transcript variant 3, mRNA] in GenBank.

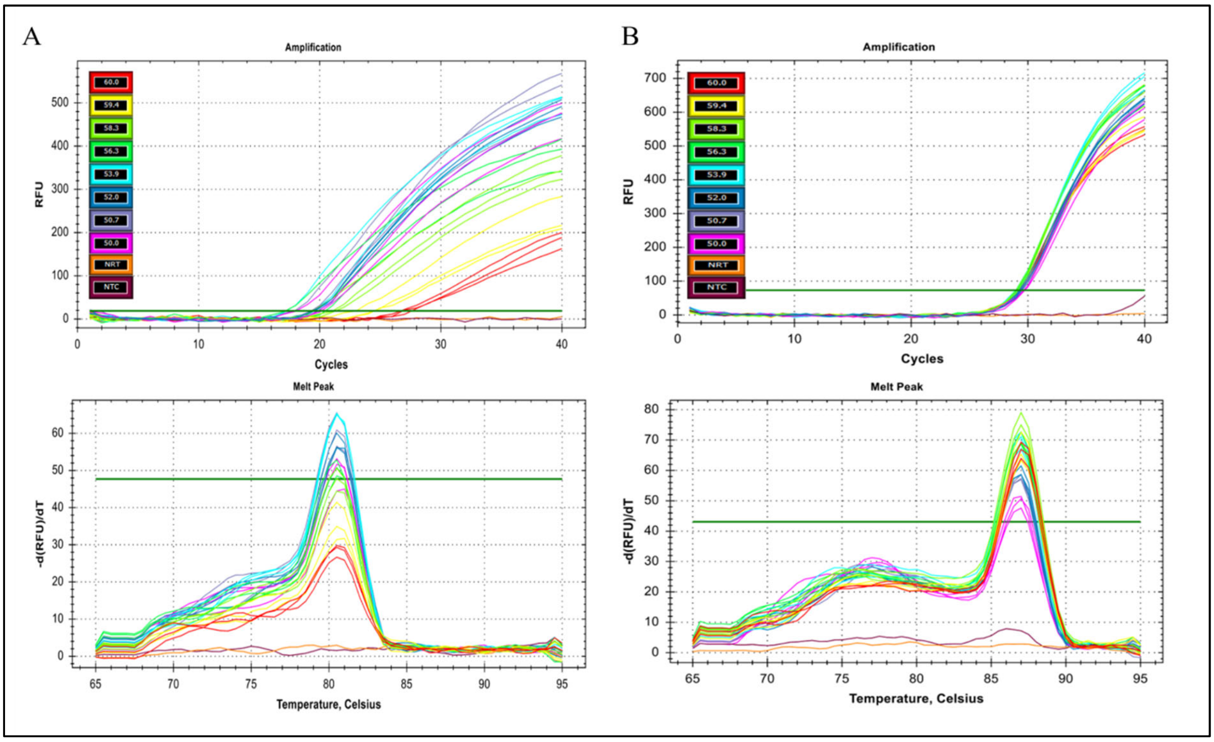

Supp Fig. 4 Annealing temperature optimization of RT-qPCR primer pairs and melt curves displaying the melting temperature (Tm) as peaks for (A) hERG1a and (B) hERG1b. The optimal amplification for hERG1a and hERG1b occur at the annealing temperatures between 50°C and 53.9°C, and 53.9°C and 58.3°C respectively. The T_m for hERG1a expression was 80.5°C and the Tm for hERG1b expression was 87°C. 53.9°C was used as the optimum annealing temperature for both hERG1a and hERG1b primers in subsequent experiments.

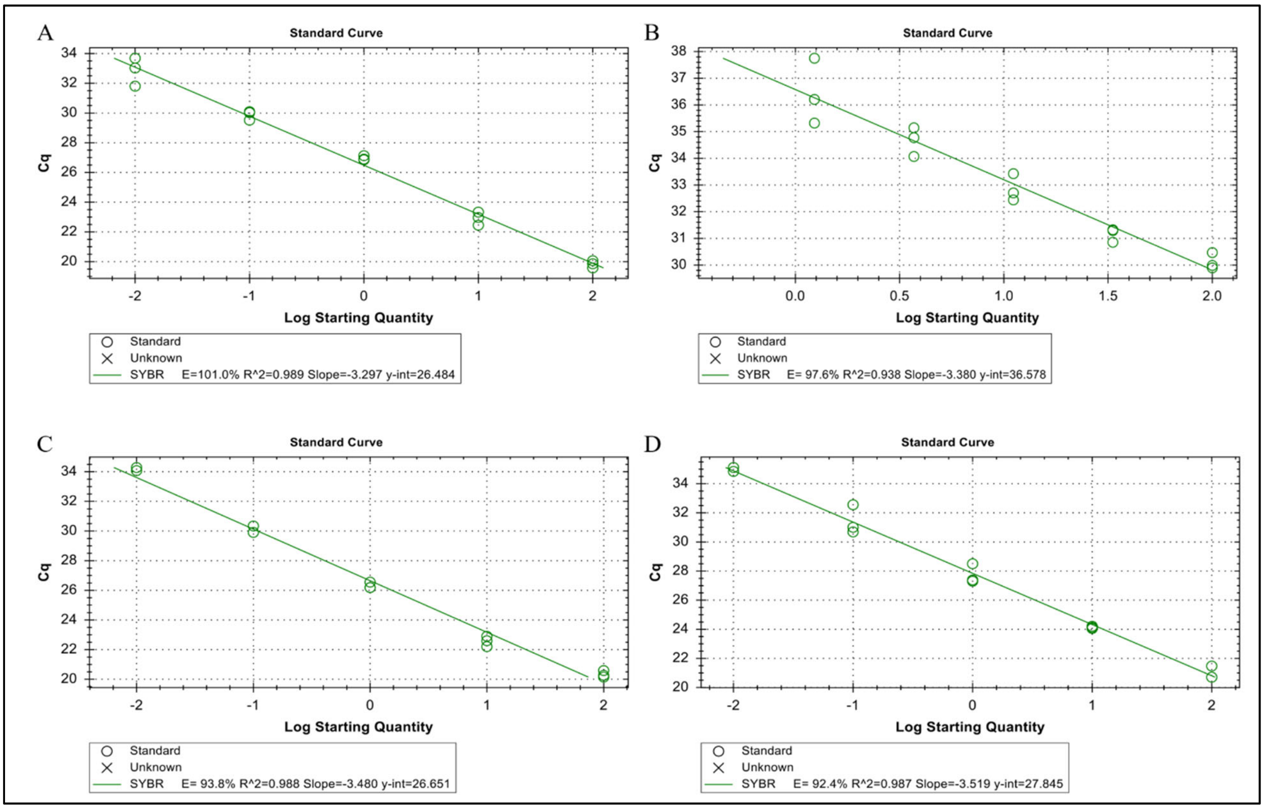

Supp Fig. 5 Standard curve plots for amplification efficiency of (A) hERG1a, (B) hERG1b, (C) ACTB and (D) GAPDH. PCR efficiency, E was determined using the formula E = 10-1/slope with data points covering a log dilution range. Data was calculated automatically using CFX Manager software version 3.1 (Bio-Rad Laboratories Inc., USA). The calculated amplification efficiency was (A) 101%, (B) 97.6%, (C) 93.8% and (D) 92.4% respectively.

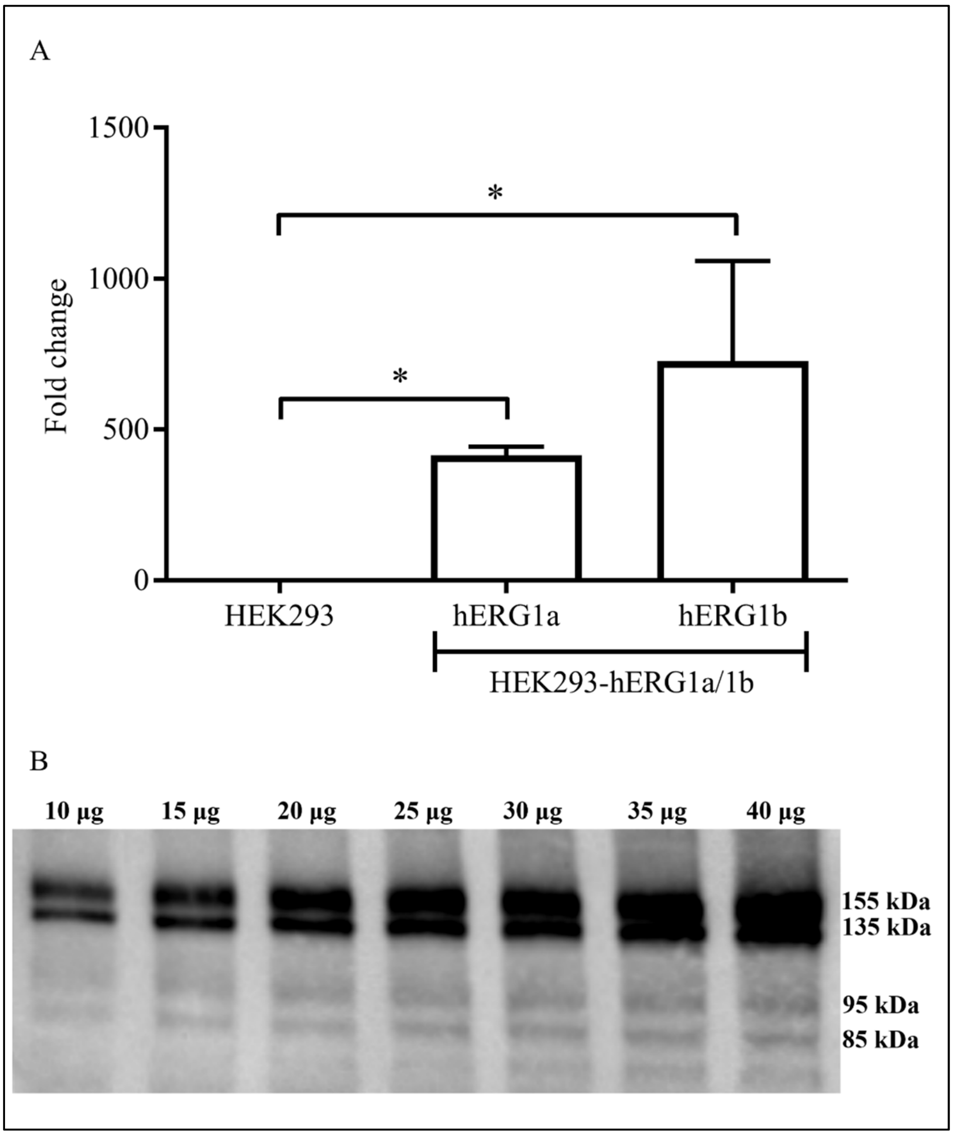

Supp Fig. 6 (A) Fold change of hERG1a and hERG1b mRNA expression in HEK293-hERG1a/1b cells relative to HEK293. Data are represented as mean replicates ± SD of 6 independent experiments ($n = 6$). Statistical significance was analyzed using unpaired t-test. *$p < 0.05$ compared to HEK293. The mRNA expression of hERG1a and hERG1b in transfected HEK293 cell were approximately 400-fold ($p < 0.0001$) and 700-fold ($p = 0.0003$) higher than untransfected HEK293 (B) A representative image showing the Western blot analysis for the hERG1a (155 kDa and 135 kDa) and hERG1b (95 kDa and 80 kDa) protein expression in HEK293-hERG1a/1b cells.

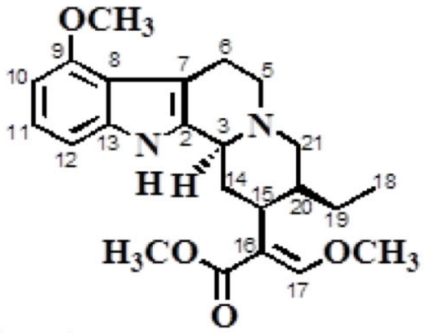

Mitragynine

1H-NMR (400 MHz, CDCl3): δ 7.73 (1H,br-s,NH), 7.43 (1H, s, H-17), 6.99 (1H, t, J=8.0Hz, H-11), 6.90 (1H, d, J=8.0Hz, H-12), 6.45 (1H, d, J=8.0Hz, H-10), 3.87 (3H, s, 9-OCH3), 3.73 (3H, s, 17-OCH3), 3.71 (3H, s, COOCH3), 3.15 (1H,br-dd, J=11.0,2.0Hz,H-3), 3.11 (1H, td, J=12.0, 2.7Hz, 3.03 (1H, dt, J=12.0, 3.6Hz, 2.93-2.99 (3H, m), 2.53 (2H, m), 2.45 (1H,dd,J=11.2, 2.8/2.9 Hz), 1.79 (2H, m), 1.62 (1H, m), 1.20 (1H, m) 0.87 (3H,t,J=7.3Hz, 18-CH3. 13C- NMR (125 MHz, CDCl3): δ 169.3 (COOCH3), 160.6 (C-17), 154.5 (C-9), 137.2 (C-13), 133.7 (C-2), 121.8 (C-11), 117.7 (C-8), 111.5 (C-16), 107.9 (C-7), 104.2 (C-12), 99.7 (C-10), 61.6 (17-OCH3), 61.3 (C-3), 57.8 (C-21), 55.3 (9-OCH3), 53.8 (C-5), 51.4 (COOCH3), 40.7 (C-20), 39.9 (C-15), 29.9 (C-14), 23.9 (C-6), 19.1 (C-19), 12.9 (C-18).

Supp Fig 7 The structure and identity of the mitragyine was confirmed using 1H-NMR and 13C-NMR analysis.

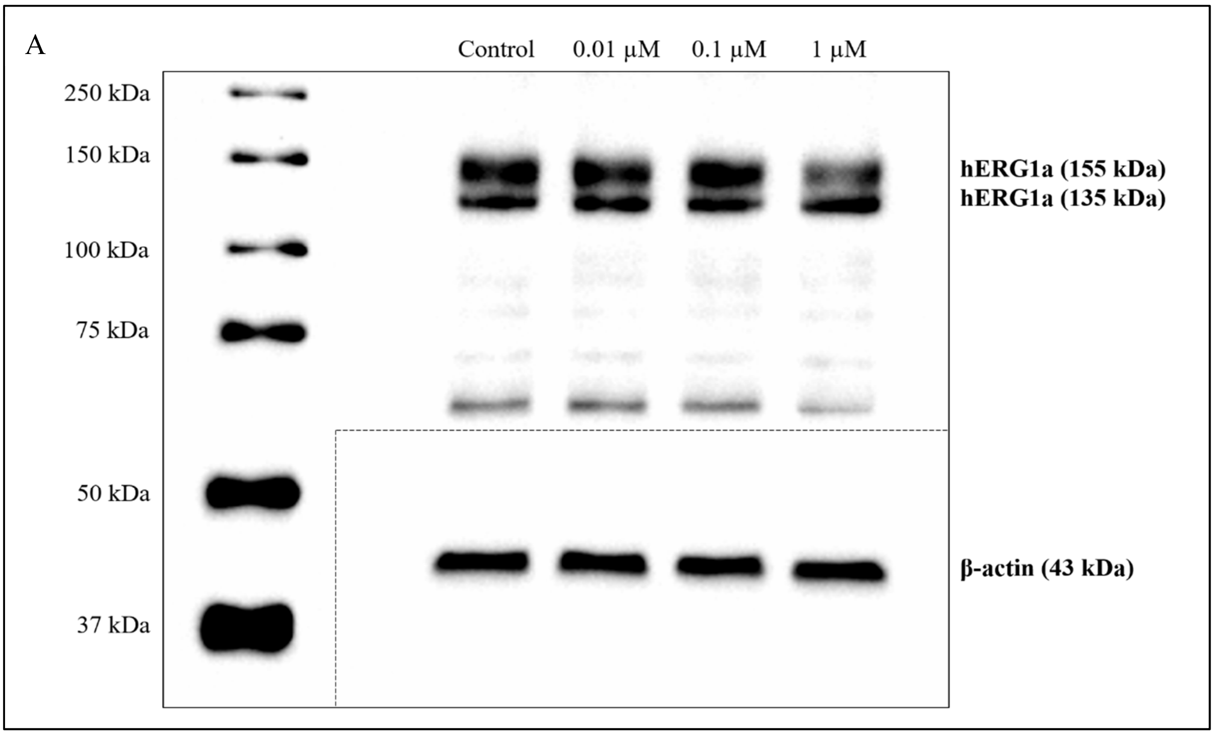

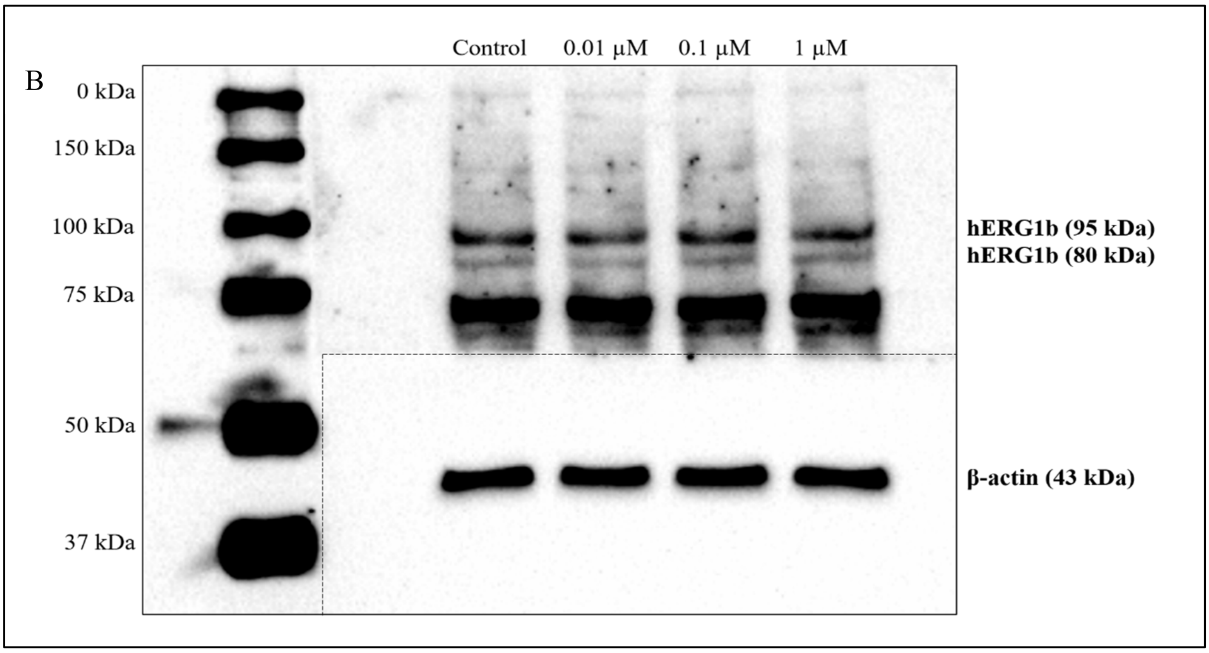

Supp Fig 8 Full-length blots for Fig. 5B. The effects of arsenic trioxide on the (A) hERG1a and (B) hERG1b protein expression. Electrophoresis was carried out at 150 V for 90 min and the protein bands were then transferred to a single PVDF membrane at 1.0A and 25 V for 30 min. The protein of interest and the internal control of protein loading, β-actin (43 kDa), were separated by cutting the membrane at around 50 kDa using the molecular weight marker as reference. The separated membranes were then placed in different containers and incubated with respective antibodies. After immunoblotting, the separated membranes were placed together and visualized using ChemiDoc™ XRS Imaging System (Bio-Rad Laboratories Inc, USA). For hERG1a & β-actin, the exposure time was 24 s, meanwhile for hERG1b & β-actin, the exposure time was 111 s.

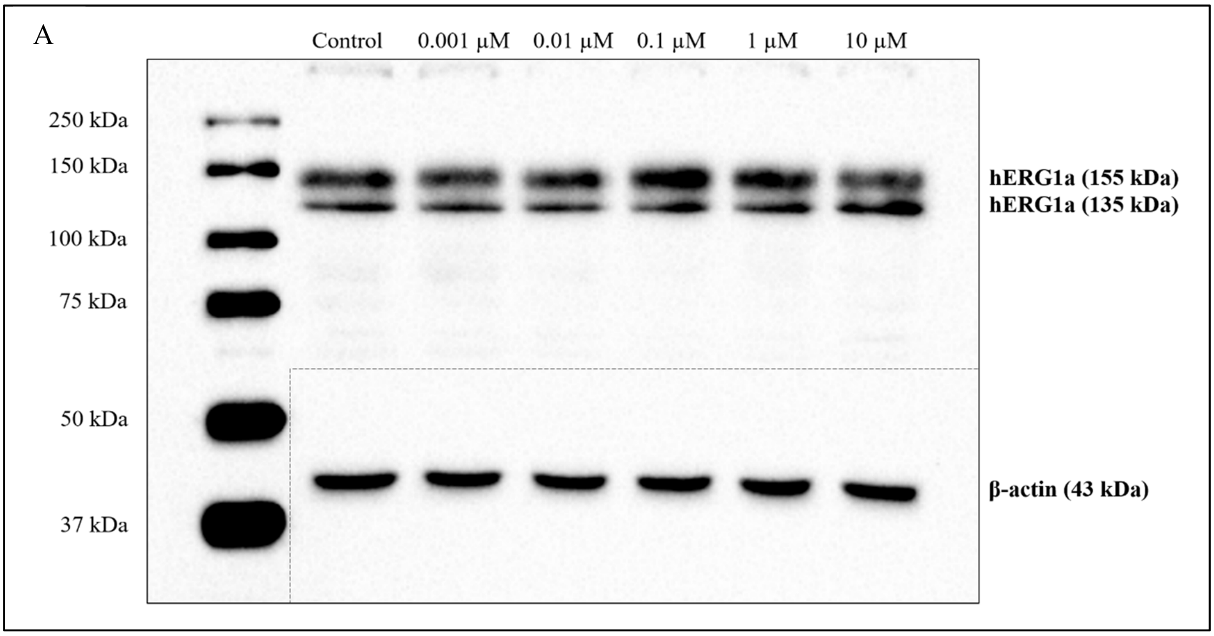
A
Control
0.001 µM
0.01 µM
0.1 µM
1 µM
10 µM
250 kDa
150 kDa
100 kDa
75 kDa
50 kDa
37 kDa
hERG1a (155 kDa)
hERG1a (135 kDa)
β-actin (43 kDa)

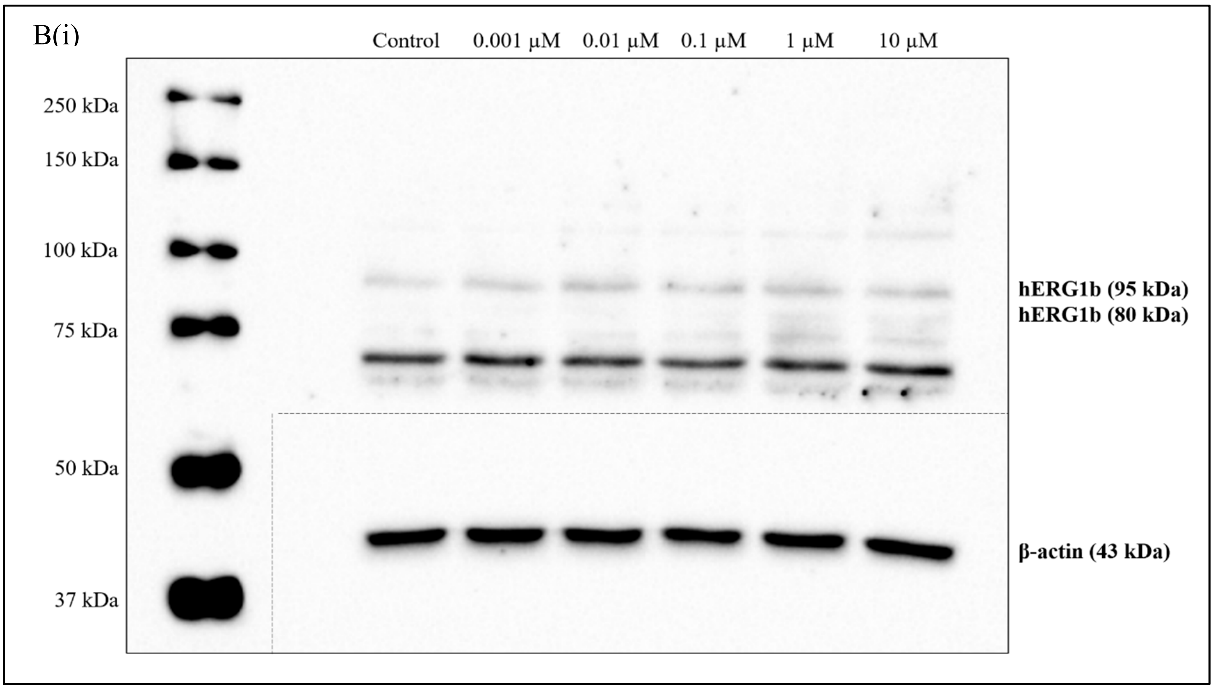
B(i)
Control
0.001 µM
0.01 µM
0.1 µM
1 µM
10 µM
250 kDa
150 kDa
100 kDa
75 kDa
50 kDa
37 kDa
hERG1b (95 kDa)
hERG1b (80 kDa)
β-actin (43 kDa)

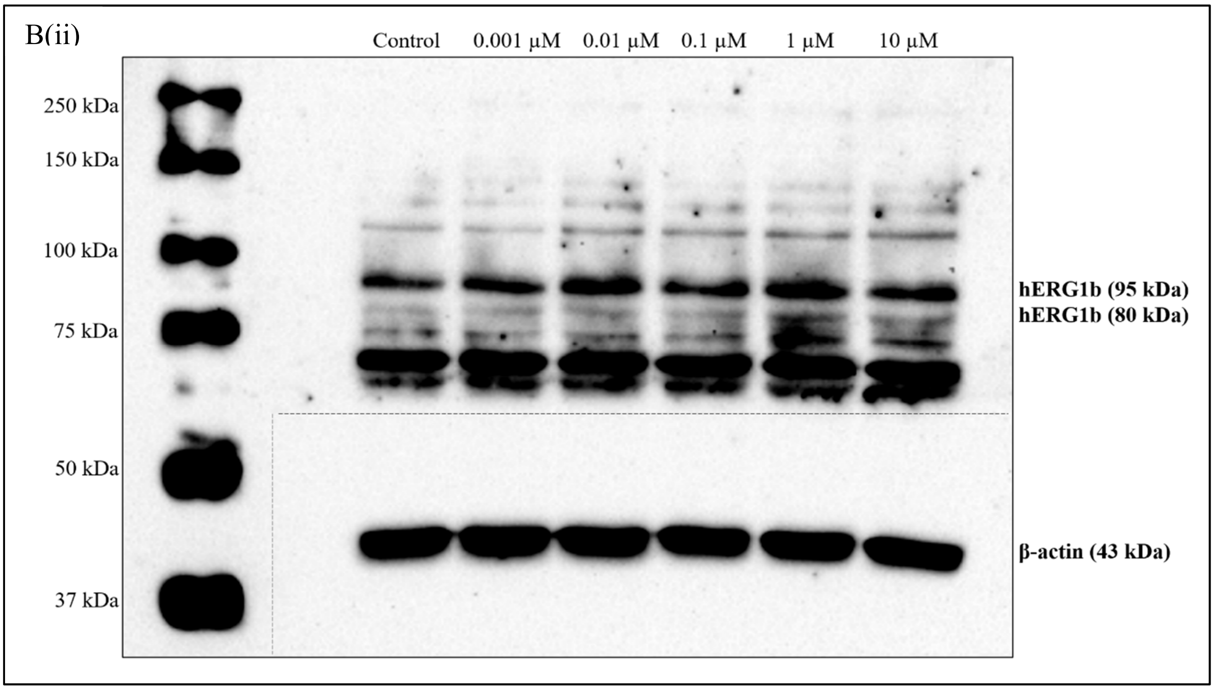

Supp Fig 9 Full-length blots for Fig. 5D. The effects of mitragynine on the (A) hERG1a and (B) hERG1b protein expression. Electrophoresis was carried out at 150 V for 90 min and the protein bands were then transferred to a single PVDF membrane at 1.0A and 25 V for 30 min. The protein of interest and the internal control of protein loading, β-actin (43 kDa), were separated by cutting the membrane at around 50 kDa using the molecular weight marker as reference. The separated membranes were then placed in different containers and incubated with respective antibodies. After immunoblotting, the separated membranes were placed together and visualized using ChemiDoc™ XRS Imaging System (Bio-Rad Laboratories Inc, USA). For hERG1a & β-actin, the exposure time was 16 s meanwhile β-actin bands were detected with exposure time of (i) 32 s, whereas hERG1b bands were detected with exposure time of (ii) 206 s.

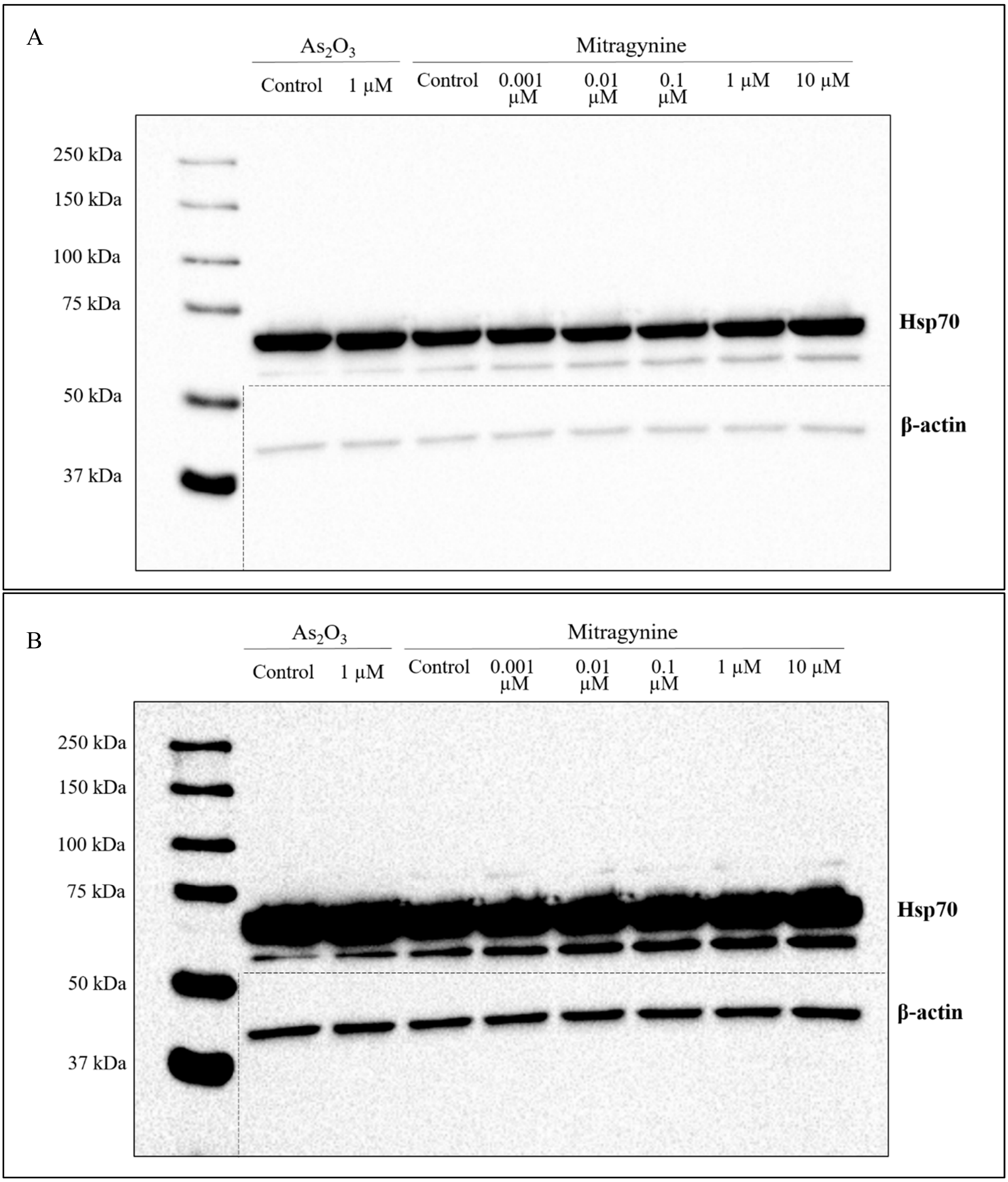

Supp Fig 10 Full-length blots for Fig. 7A. The effects of arsenic trioxide and mitragynine on the protein expression of Hsp70 proteins. Electrophoresis was carried out at 150 V for 90 min and the protein bands were then transferred to a single PVDF membrane at 1.0A and 25 V for 30 min. The protein of interest and the internal control of protein loading, β-actin (43 kDa), were separated by cutting the membrane at around 50 kDa using the molecular weight marker as reference. The separated membranes were then placed in different containers and incubated with respective antibodies. After immunoblotting, the separated membranes were placed together and visualized using ChemiDoc™ XRS Imaging System (Bio-Rad Laboratories Inc, USA). Hsp70 bands were detected with exposure time of (A) 1.5 s, whereas β-actin bands were detected with exposure time of (B) 10 s.

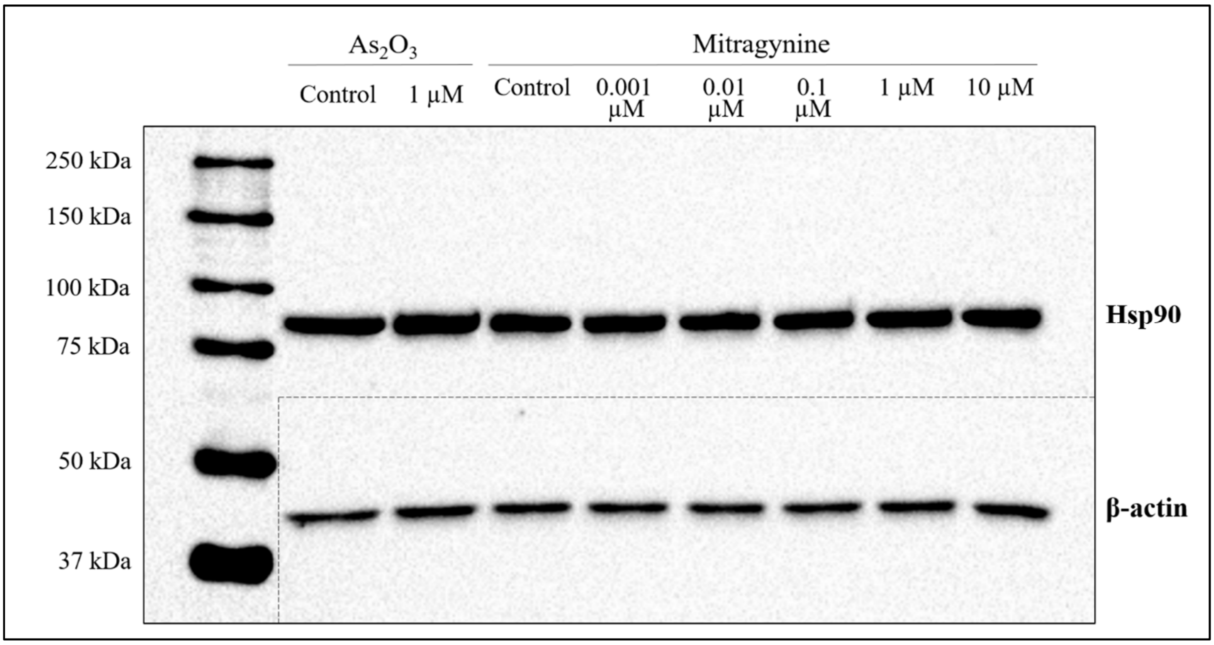

Supp Fig 11 Full-length blot for Fig. 7B. The effects of arsenic trioxide and mitragynine on the protein expression of Hsp90 proteins. Electrophoresis was carried out at 150 V for 90 min and the protein bands were then transferred to a single PVDF membrane at 1.0A and 25 V for 30 min. The protein of interest and the internal control of protein loading, β-actin (43 kDa), were separated by cutting the membrane at around 50 kDa using the molecular weight marker as reference. The separated membranes were then placed in different containers and incubated with respective antibodies. After immunoblotting, the separated membranes were placed together and visualized using ChemiDoc™ XRS Imaging System (Bio-Rad Laboratories Inc, USA). Both Hsp90 & β-actin was detected with exposure time of 7.6 s.

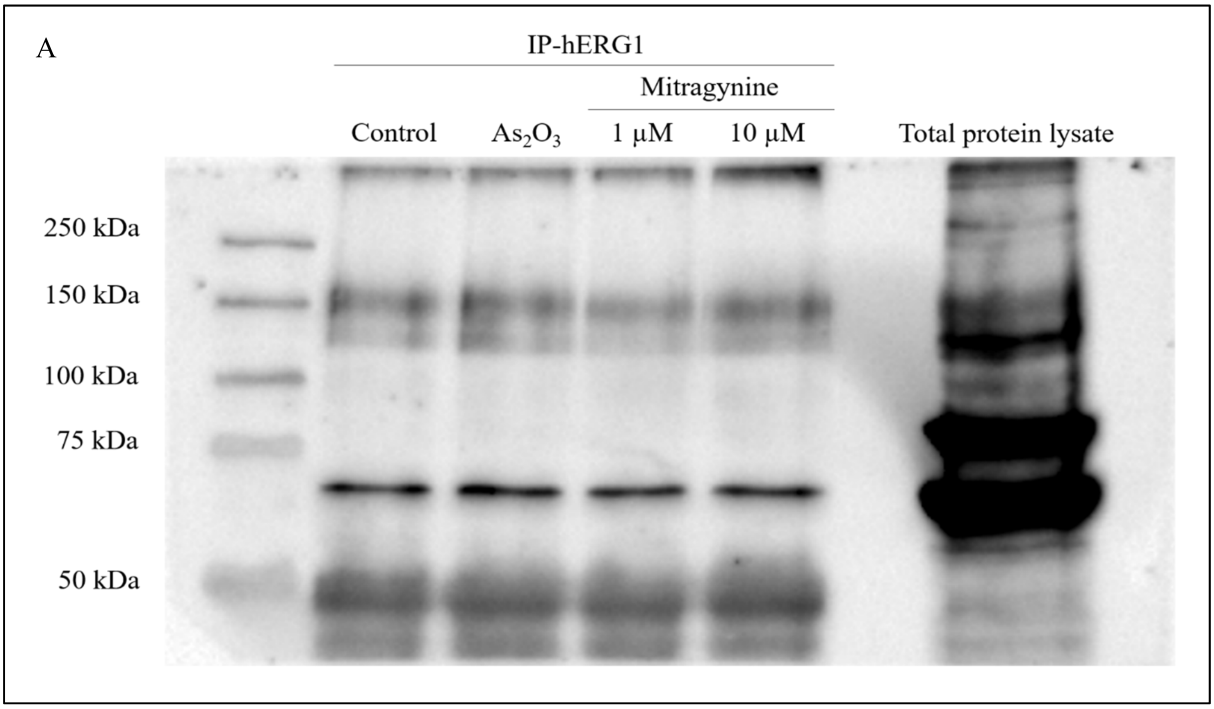

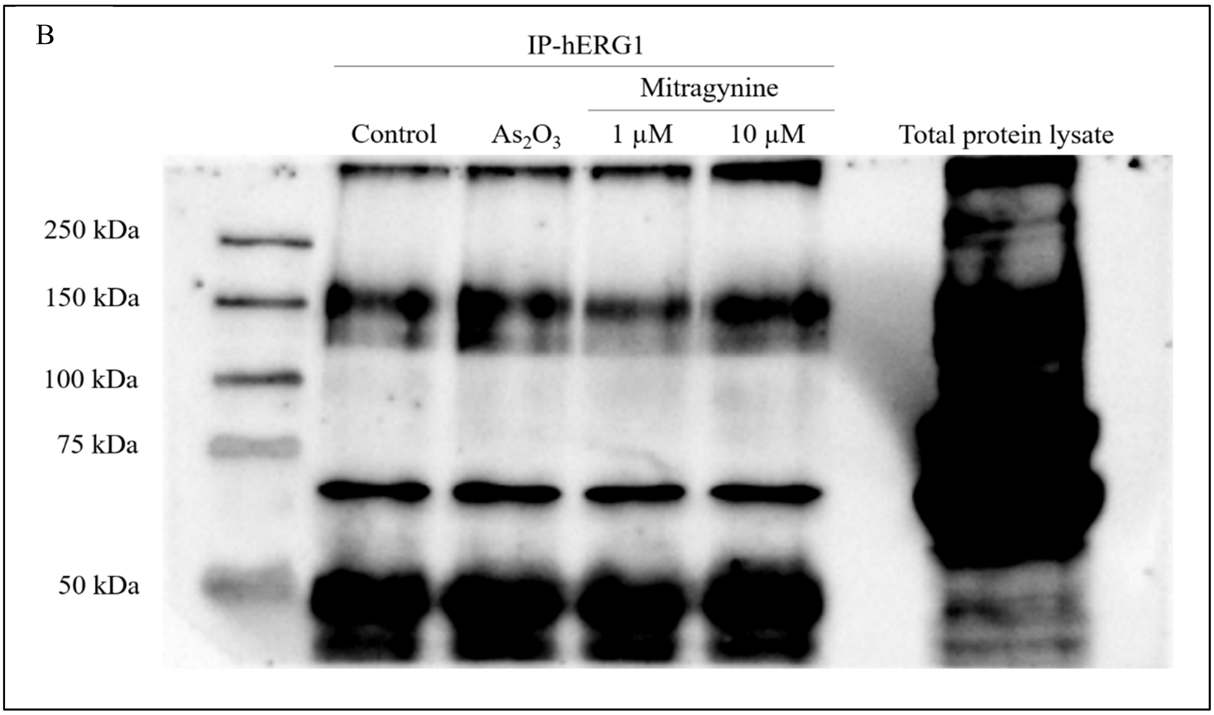

Supp Fig 12 Full length blots for Fig. 7C. The effects of arsenic trioxide and mitragynine on the interaction between cg-hERG1a with Hsp70. Total protein lysates were shown in the blot with exposure time of 5 s (A). For IP-hERG1a, the proteins of interest were shown in the blot with exposure time of 11 s (B).

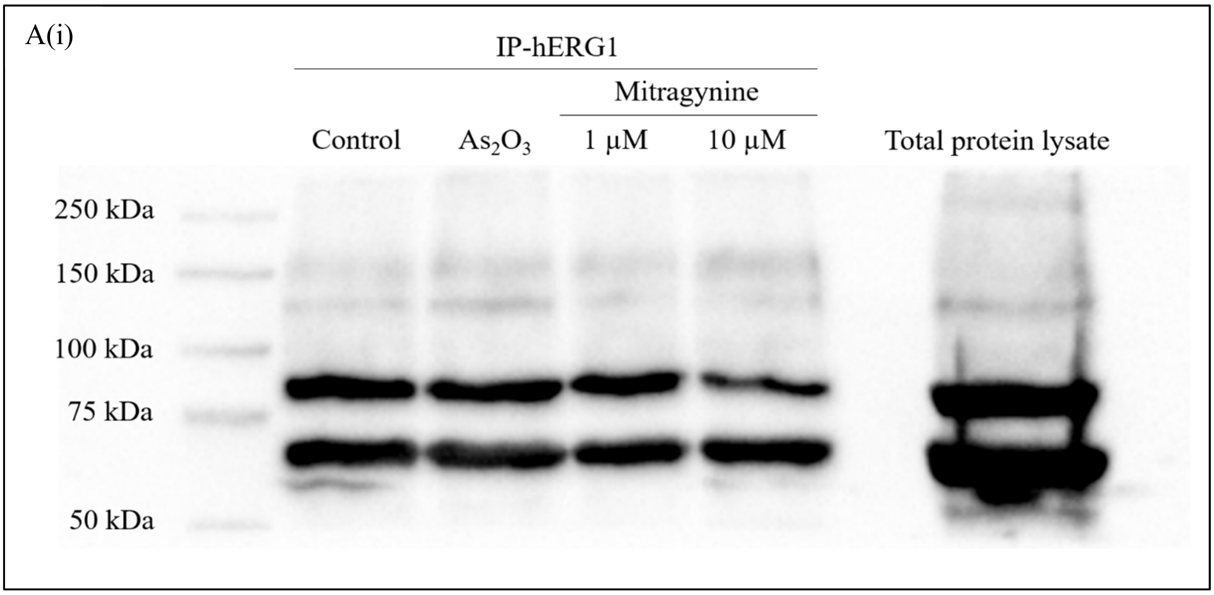
A(i)
IP-hERG1
Mitragynine
Control
As_2O_3
1 µM
10 µM
Total protein lysate
250 kDa
150 kDa
100 kDa
75 kDa
50 kDa

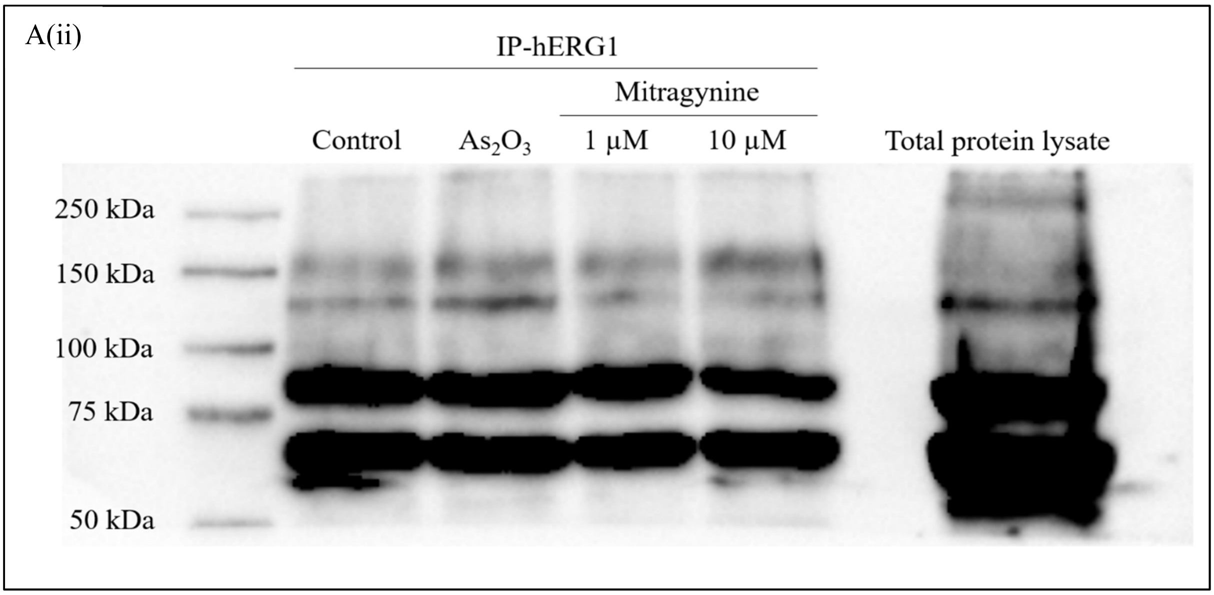
A(ii)
IP-hERG1
Mitragynine
Control
As_2O_3
1 µM
10 µM
Total protein lysate
250 kDa
150 kDa
100 kDa
75 kDa
50 kDa

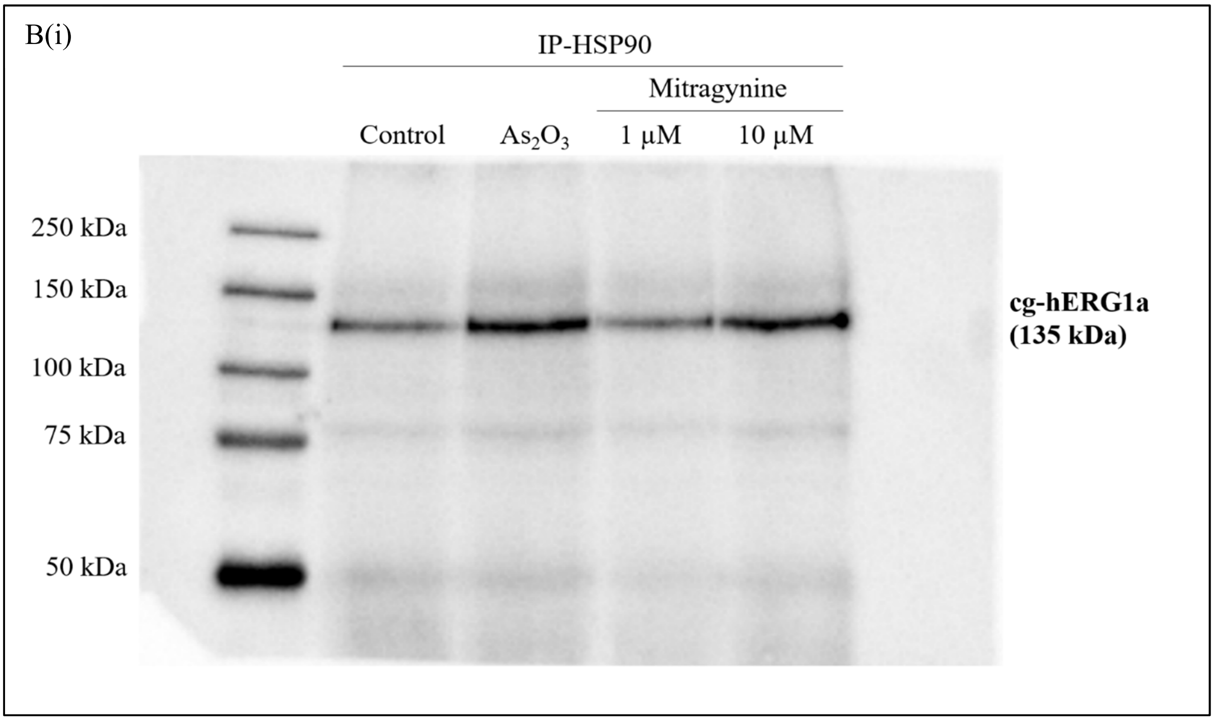
B(i)
IP-HSP90
Mitragynine
Control
As_2O_3
1 µM
10 µM
250 kDa
150 kDa
100 kDa
75 kDa
50 kDa
cg-hERG1a
(135 kDa)

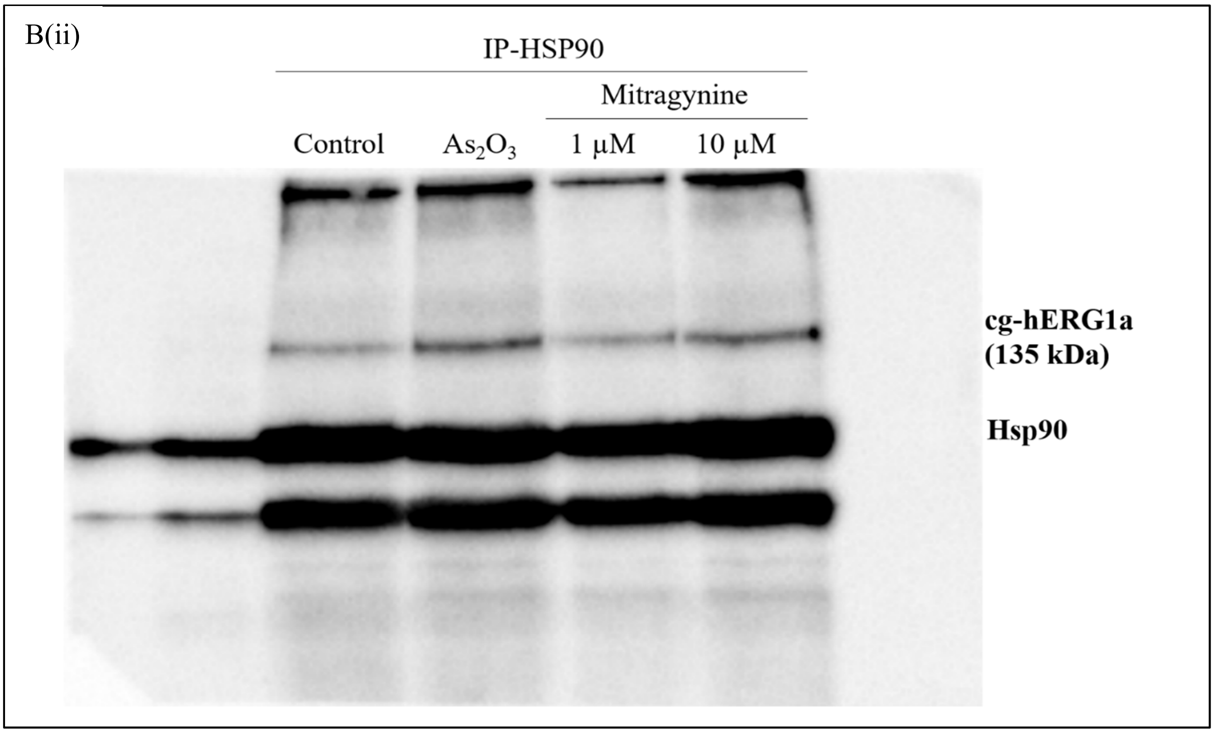

Supp Fig 13 Full length blots for Fig. 7D. The effects of arsenic trioxide and mitragynine on the interaction between cg-hERG1a with Hsp90. (A) For IP-hERG1a, blots with different exposure times (i) 1 s and (ii) 8 s were shown. (B) For IP-Hsp90, the blot was first incubated with antibody detecting (i) hERG1 or (ii) Hsp90 and visualized using ChemiDoc™ XRS Imaging System. The blots were viewed with exposure time of 1s.
